# 3D Engineered Dual‐Redox Zinc‐Iodine Microbatteries for Intrinsically Safe on‐Chip Energy Storage

**DOI:** 10.1002/advs.75453

**Published:** 2026-04-27

**Authors:** Nibagani Naresh, Sanat Nalini Paltasingh, Yijia Zhu, Xiaopeng Liu, Yujia Fan, Monojit Mondal, Su Jin Heo, Shuhui Li, Mingqing Wang, Shaoliang Guan, Yanzhuo Li, Georgios Nikiforidis, Shahab Akhavan, Ivan P. Parkin, Saroj Kumar Nayak, Michael De Volder, Buddha Deka Boruah

**Affiliations:** ^1^ Institute For Materials Discovery University College London London UK; ^2^ School of Basic Sciences Indian Institute of Technology Bhubaneswar Khorda Odisha India; ^3^ Institute For Manufacturing Department of Engineering University of Cambridge Cambridge UK; ^4^ Department of Chemistry University College London London UK; ^5^ Department of Materials Science & Metallurgy University of Cambridge Cambridge UK

**Keywords:** 3D porous current collector, dual‐redox chemistry, on‐chip energy storage, polyaniline cathode, zinc‐iodine microbattery

## Abstract

The ongoing miniaturization of electronic technologies—from medical implants and microrobots to IoT and sensor networks—demands compact, intrinsically safe, and high‐energy‐density microbatteries (MBs). Yet, achieving on‐chip energy storage that simultaneously delivers high capacity, rapid kinetics, and scalability remains a formidable challenge. Here, we report advance zinc‐iodine (Zn//I_2_) MBs that exploit a synergistic dual‐redox chemistry by introducing ZnI_2_ into a Zn(CF_3_SO_3_)_2_ gel electrolyte, enabling reversible I^−^/I_3_^−^ conversion in tandem with Zn^2^^+^ plating/stripping. Coupled with a polyaniline (PANI) micro‐cathode, zinc micro‐anode, and 3D porous Au interdigitated current collector, this architecture delivers over 26‐fold enhancement in charge storage relative to conventional Zn‐ion MBs using identical electrodes. The optimized Zn//I_2_ MBs achieve an improved areal capacity of 314 µAh cm^−^^2^, energy density of 363 µWh cm^−^^2^, and power density of 5385 µW cm^−^^2^. Density functional theory (DFT) calculations and electrochemical analyses reveal strong I^−^/I_3_^−^ adsorption on PANI, confirming its superior redox hosting capability and hybrid charge storage behavior. This work establishes a new design paradigm for intrinsically safe, and CMOS‐compatible Zn‐based MBs, offering a transformative pathway toward on‐chip powered, fully integrated microsystems and next‐generation smart electronics.

## Graphical abstract

Dual‐redox Zn//I_2_ microbatteries enable high‐performance on‐chip energy storage by combining Zn^2^^+^ plating/stripping with reversible I^−^/I_3_^−^ chemistry. Integrated with PANI micro‐cathode and 3D porous Au collectors, the system delivers enhanced capacity, energy, and power, advancing safe, CMOS‐compatible microsystems for next‐generation electronics.

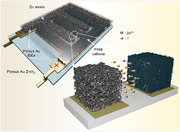

## 1 Introduction

The rapid advancement in the miniaturization of microelectronic technologies has enabled the development of microsensors, IoT devices, medical implants, and microrobots. To operate seamlessly and realize systems‐on‐a‐chip, these devices demand compatible energy storage systems that not only provide high energy and power densities but also offer long‐term cycling stability and, importantly, safety—especially for applications such as medical implants. As a result, considerable effort has been devoted to the development of high energy density MBs, particularly those based on lithium‐ion and sodium‐ion chemistries, to meet the stringent energy demands of on‐chip microdevices [1, 2, 3, 4]. These MBs are capable of delivering the required energy for efficient operation. However, some applications require not only high power but also high safety, a demand that lithium‐ion and sodium‐ion systems need improving [5, 6]. This is mainly due to the use of toxic and flammable organic electrolytes, which complicate the fabrication process and hinder on‐chip integration with microelectronics [7, 8]. In contrast, aqueous zinc‐ion battery systems have emerged as a promising alternative due to their inherent safety, cost‐effectiveness, and ease of fabrication [9, 10]. Typically, these batteries utilize cathode materials such as metal oxides (e.g., vanadium‐based, manganese‐based) [11, 12, 13, 14], Prussian blue analogs [15], or conductive polymers (e.g., polyaniline (PANI), polypyrrole) [16, 17, 18], paired with zinc (Zn)‐based anodes. [19, 20] The Zn anode offers a high theoretical capacity of 820 mAh/g and a high volumetric capacity (5855 mAh/cm^3^) [21, 22]. However, many commonly used cathode materials still suffer from limited capacities—often below 500 mAh/g—which becomes a significant limitation, particularly in MB applications where the device footprint is extremely constrained. Therefore, boosting the capacity of cathode materials is critically important for MB development. Enhancing material loading on microelectrodes and adopting effective integration strategies are essential steps toward achieving high‐performance microscale energy storage systems.

One of the most effective strategies for advancing high‐performance MBs lies in the development of micro‐scale porous current collectors that enable efficient loading of active battery materials. Unlike conventional sandwich‐type electrodes used in coin or pouch cells, MBs typically avoid the use of conductive additives and polymeric binders due to the limited device footprint and challenges in integrating these components onto micro‐scale current collectors [23, 24]. However, this often results in limited electrical conductivity. Hence, simply increasing the mass loading of cathode materials can then compromise rate capability. To overcome these challenges, the design of porous metallic current collectors has proven highly beneficial. Such architectures enhance electron transport pathways and offer a robust scaffold for the effective loading of active materials, enabling improved energy storage performance without sacrificing rate capability. In our recent work, we demonstrated how 3D porous gold (Au) and copper current collectors significantly enhance performance when the same active materials—Zn anodes and PANI cathodes—are used, compared to flat Au interdigitated electrodes (IDEs) [25, 26, 27]. These porous architectures allow for greater material utilization and improved ion/electron transport, thereby increasing the areal energy density of MBs. Despite these advances, most reported MBs still deliver modest areal energies—typically below 50 µWh/cm^2^. This highlights the need for further innovation to realize the full potential of microscale energy storage systems.

In this work, we report a significant advancement in the performance of Zn‐based MBs through strategic electrode design and dual‐electrolyte engineering. By loading PANI and Zn onto 3D porous Au scaffolds and incorporating a dual‐ion electrolyte—achieved by adding ZnI_2_ into a Zn(CF_3_SO_3_)_2_‐based gel electrolyte commonly used in zinc‐ion MBs (ZIMBs)—we demonstrate remarkable improvements in both energy storage capacity and rate capability using the same electrode materials of PANI cathode and Zn anode. This system harnesses the reversible two‐electron redox reaction of iodide/triiodide (I^−^/I_3_^−^) chemistry, which not only enhances the capacity but also enables high‐rate operation with stable performance even at areal currents up to 5 mA cm^−^^2^. The optimized MB system delivers an areal capacity of 315 µAh cm^−^^2^, an areal energy density of 363 µWh cm^−^^2^, and an impressive areal power density of 5385 µW cm^−^^2^. Comprehensive electrochemical characterization and density functional theory (DFT) calculations of I^−^/I_3_^−^ adsorption on the PANI cathode reveal that PANI serves as a more efficient I^−^/I_3_^−^ host compared to various carbon‐based materials. Further in‐depth analysis confirms a hybrid charge storage mechanism, which is instrumental in achieving this outstanding performance. These findings pave the way for the next generation of high‐performance, intrinsically safe MBs suitable for a wide range of miniaturized electronic applications.

## 2 Results and Discussion

**FIGURE 1:**
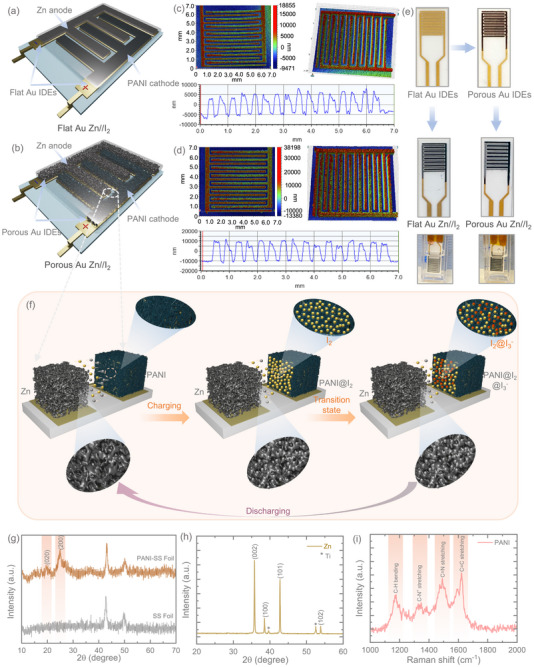
(a,b) Schematic representations of the fabrication processes for flat Au Zn//I_2_ MB (or ZIMB) and porous Au Zn//I_2_ MB devices, illustrating the electrodeposition of the PANI cathode and Zn anode onto the flat and porous Au IDE current collectors. (c,d) 3D and 2D profilometry images of flat and porous Au Zn//I_2_ MBs, with corresponding height profiles shown below. The electrode thicknesses are approximately 6 µm and 16 µm, respectively, excluding the ∼4 µm thickness of the underlying flat Au IDEs. (e) Digital photographs of the flat Au IDE (top left), porous Au IDE (top right), flat Au Zn//I_2_ MB (bottom left), and porous Au Zn//I_2_ MB (bottom right). The bottom panel shows the assembled devices immersed in gel electrolytes. (f) Schematic illustration of the charge storage mechanism in the Zn//I_2_ MBs during charging and discharging processes. (g,h) XRD patterns of stainless steel (SS) foil and electrodeposited PANI and Zn on titanium (Ti) substrates, using the same deposition conditions as those applied to the MBs. (i) Raman spectrum of electrodeposited PANI.

Figure 1a,b shows schematic illustrations of our MBs, which were fabricated by electrodepositing the active Zn and PANI materials onto both planar and 3D porous Au IDEs. When tested in a Zn(CF_3_SO_3_)_2_ gel electrolyte, these devices are referred to as ZIMBs. Upon the addition of ZnI_2_ into the Zn(CF_3_SO_3_)_2_ gel electrolyte, the devices are referred to as flat Au Zn//I_2_ MBs (for materials loaded onto flat Au IDEs) and porous Au Zn//I_2_ MBs (for those on porous Au IDEs). The inclusion of ZnI_2_ dramatically alters the charge storage mechanism by introducing a redox couple of I^−^/I_3_^−^ into charge storage chemistry. Although elemental I_2_ is not used directly as a cathode material, the electrolyte serves as an iodide (I^−^) source, effectively transforming the system into a Zn‐iodine battery in terms of electrochemical behavior. The Au IDEs are patterned onto a ceramic substrate with 200 µm width and gap. To enhance the efficient loading of active materials within the constrained device area, a 3D porous Au metal scaffold was electrodeposited onto the flat Au IDEs using the dynamic hydrogen bubble template method, described in detail in the Experimental Section of the Supporting Information [27]. This method generates hydrogen bubbles that act as soft templates during electrodeposition, enabling the formation of a highly porous Au scaffold. The PANI cathode and Zn anode were subsequently electrodeposited onto both flat and porous Au IDEs. The electrodeposition approach facilitates binder‐free and additive‐free integration of the active materials onto the electrodes, ensuring efficient and uniform loading. Detailed procedures for the electrodeposition of both PANI and Zn are provided in the Supporting Information. Figure 1c,d show 2D and 3D profilometry images of the flat and porous Au Zn//I_2_ MBs (or ZIMBs). The height profiles indicate that the flat MBs exhibit an approximate electrode thickness of 6 µm, whereas the porous MBs reach 16 µm—excluding the baseline 4 µm thickness of the flat Au IDEs. This confirms that the dynamic hydrogen bubble template method significantly enhances the active material within the limited 0.36 cm^2^ device area. Additionally, the profilometry images demonstrate uniform deposition of both the PANI cathode and Zn anode across the IDEs. Figure 1e presents digital photographs of the flat and porous Au IDEs, the corresponding assembled MB devices, and the devices immersed vertically in clear, transparent gel electrolytes. These include 2 m Zn(CF_3_SO_3_)_2_ in PVA for Zn‐ion battery chemistry and 2 m Zn(CF_3_SO_3_)_2_ + 0.2 m ZnI_2_ in PVA for Zn‐iodine battery chemistry. As shown in Figure 1f, upon introducing ZnI_2_ into the 2 m Zn(CF_3_SO_3_)_2_ electrolyte to incorporate the I^−^/I_3_^−^ redox couple into the charge storage chemistry, the possible charge storage mechanism proceeds as follows. During charging, Zn^2^^+^ ions gain electrons and are reduced to metallic Zn at the anode (Zn^2^^+^ + 2e^−^ → Zn), while I^−^ ions within the PANI lose electrons to form solid I_2_ (2I^−^ → I_2_ + 2e^−^). The generated I_2_ subsequently reacts with excess I^−^ to form soluble I_3_^−^ species (I_2_ + I^−^ → I_3_^−^). During discharge, the Zn anode undergoes stripping to release Zn^2^^+^ ions, while the I_3_^−^ species are reduced back to I^−^ at the cathode, thereby completing the reversible electrochemical cycle [28]. Figure 1g,h shows the XRD patterns of electrodeposited PANI and Zn, respectively. Due to the limited footprint of the MBs, materials were electrodeposited onto stainless steel (SS) foil and Ti foil under identical conditions for XRD analysis. The XRD pattern of PANI (Figure 1g), electrodeposited on SS foil, exhibits broad diffraction peaks at 2*θ* ≈ 20.8° and 25.9°, corresponding to the (020) and (200) planes, respectively, along with additional peaks arising from the SS substrate [29], indicating crystalline regions within an otherwise amorphous matrix. The XRD pattern of Zn (Figure 1h) shows sharp peaks at 2*θ* around 36.53°, 39.26°, 43.28°, and 54.56°, which correspond to the (002), (100), (101), and (102) planes, respectively [30]. The Raman spectrum of PANI (Figure 1i) demonstrates prominent peaks at 1169, 1330, 1490, and 1617 cm^−1^, which correspond to the C─H bending, C─N^+^ stretching, C═N stretching, and C═C stretching modes, respectively [31].

**FIGURE 2:**
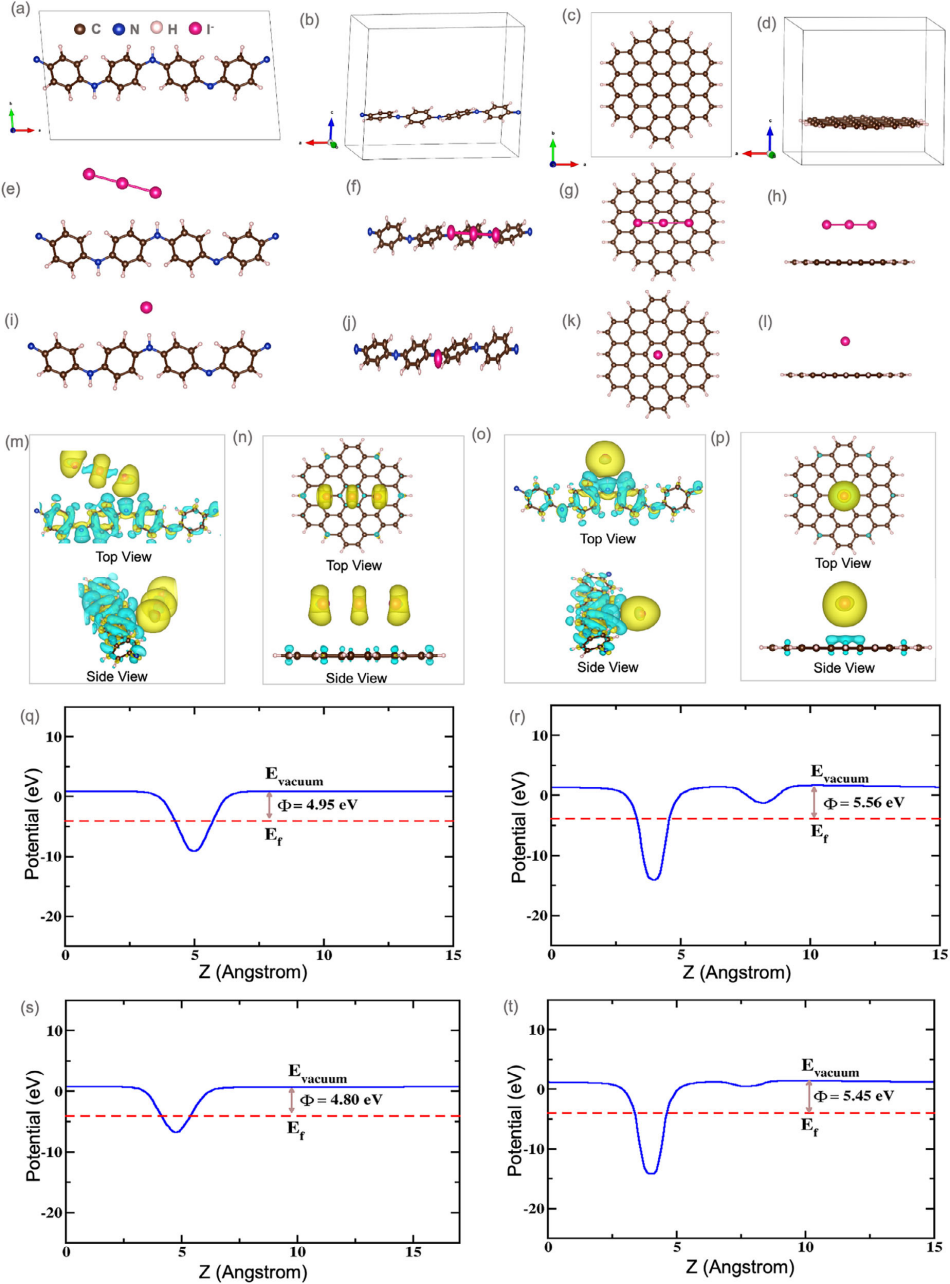
Optimized structures of (a,b) PANI: top view and side view, and (c,d) 19‐ring AC: top view and side view, respectively. Optimised structure of I_3_^−^ adsorbed on PANI (e), Top view, (f) Side view, and on AC (g) Top view, (h) Side view, respectively. Optimised structure of I^−^ adsorbed on PANI (i) Top view, (j) Side view, and on AC (k) Top view, (l) Side view, respectively. The dark grey, blue, off‐white, and pink spheres represent carbon, nitrogen, hydrogen, and iodine atoms, respectively. Charge density difference (CDD) plot for (m) I_3_^−^ adsorbed on PANI, (n) I_3_^−^ adsorbed on AC, (o) I^−^ adsorbed on PANI, and (p) I^−^ adsorbed on AC. The yellow color represents the charge accumulation region, while the cyan color indicates the charge depletion region, with an iso‐surface value of 0.0007 e/Å^3^. Workfunction plot of (q) I_3_^−^ adsorbed on PANI, (r) I_3_^−^ adsorbed on AC, (s) I^−^ adsorbed on PANI, and (t) I^−^ adsorbed on AC surfaces.

To theoretically assess the suitability of PANI as an efficient I^−^/I_3_^−^ host for realizing dual‐redox chemistry prior to experimental validation, we conducted a comprehensive density functional theory (DFT) study. The interactions between I^−^/I_3_^−^ and PANI were compared with those on commonly considered activated carbon (AC) hosts. Initially, the crystal structures of PANI and ACs were modeled, followed by the adsorption of I_3_^−^ and I^−^ species to evaluate their binding interactions. As illustrated in Figure 2a,b, the DFT‐optimized PANI structure was identified as monoclinic with lattice parameters a = 11.34Å, b = 11.31 Å, and c = 17.05 Å. The optimized bond lengths with an average (C─C: 1.40 Å, C–H: 1.09 Å, N–H: 1.01 Å, C–N: 1.35 Å, C–NH: 1.39 Å) and bond angles ∠C─C─C: 120°, ∠C─C─H: 119°, ∠C─N─C: 124°, ∠C─NH─C: 129°) were consistent with prior literature [32]. Accurate atomistic models that represent the geometrical structure and composition of ACs generally require substantial computational resources. To balance accuracy and efficiency, small polycyclic aromatic hydrocarbons such as coronene are often employed as representative AC models in theoretical studies to describe their interactions with various molecules [33, 34, 35, 36, 37]. In this work, we adopted a model of AC consisting of a 19‐ring graphene fragment terminated with hydrogen atoms (coronene: C_54_H_18_) as shown in Figure 2c,d. The optimized bond lengths and average bond angles (C─C: 1.42 Å, C─H: 1.09 Å, ∠C─C─C: 120°, ∠C─C─H: 119°) agreed with reported structures. Along with this hexagonal C‐ring form of AC, we also investigated other forms of ACs, like graphite [38] and porous graphite [39], whose optimised structures are shown in Figure S1, to compare adsorption behaviors. Upon adsorption (Figure 2e–l; Figure S2), PANI exhibited significantly stronger binding with both I_3_^−^ and I^−^ than all carbon‐based counterparts. The binding energies for I_3_^−^ on PANI, coronene, graphite, and porous graphite were −1.54, −0.29, −0.70, and −0.55 eV, respectively; for I^−^, the corresponding values were −1.31, −0.37, −0.72, and −0.59 eV, respectively. Moreover, PANI showed higher binding strength than reported other nitrogen‐doped graphitic sites such as graphene (−0.78 eV), pyrrolic N (−0.83 eV), pyridinic N (−0.86 eV), and graphitic N (−1.28 eV) [40].

To gain deeper insight into the charge redistribution behavior between I^−^/I_3_^−^ species and the host surfaces, charge density difference (CDD) analyses were performed. As illustrated in Figure 2m–p, the CDD maps reveal distinct charge depletion (cyan regions) near the host surface and charge accumulation (yellow regions) around the adsorbed I^−^/I_3_^−^ species. This indicates substantial electron transfer from the host material to the adsorbates, confirming their anionic nature (I^−^/I_3_^−^). Notably, PANI exhibits a more pronounced charge redistribution upon adsorption compared with ACs. Bader charge analysis (Table S1) further supports this observation, showing that a larger amount of charge is transferred from PANI to the I^−^/I_3_^−^ species than from ACs, corroborating the stronger interfacial interaction in the PANI system. To further elucidate the electronic characteristics of these interactions, the work function of each surface was evaluated before and after molecular adsorption. The work function reflects the minimum energy required for an electron to escape from the surface into the vacuum; thus, a lower value facilitates electron emission or injection, improving charge transfer kinetics at the cathode interface. As shown in Figure 2q–t and Figure S2e–h, adsorption of I^−^/I_3_^−^ on PANI leads to a lower work function than on ACs, consistent with enhanced electron donation and the increased electronegativity of the adsorbed species, as confirmed by Bader analysis. Collectively, these findings clearly demonstrate the stronger electronic coupling and superior adsorption affinity of PANI toward I^−^/I_3_^−^, establishing it as a more effective and efficient cathode host than AC.

**FIGURE 3:**
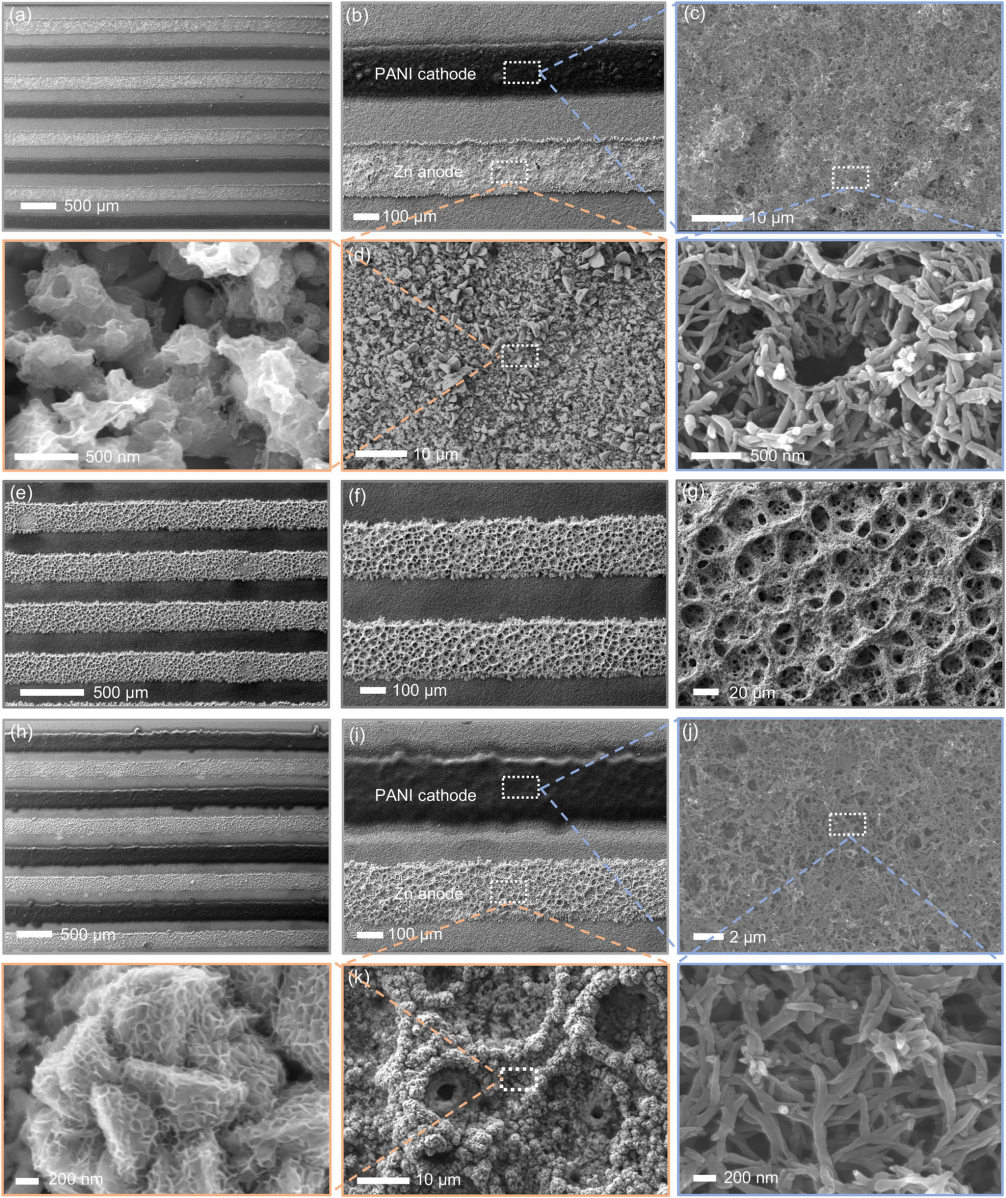
(a,b) SEM images of the flat Au Zn//I_2_ MB at different magnifications, confirming successful electrodeposition of PANI cathode and Zn anode on flat Au IDEs. (c) High‐magnification SEM image of electrodeposited PANI showing nanowire‐like morphology with distinct porosity, which enhances electrolyte interaction and interfacial contact. (d) High‐magnification SEM image of electrodeposited Zn on flat Au IDEs, revealing a mixed flake and nanosheet morphology. (e–g) SEM images of 3D porous Au scaffold IDEs at different magnifications. (h,i) SEM images of the porous Au Zn//I_2_ MB at different magnifications, demonstrating uniform and complete loading of PANI onto the 3D porous Au scaffold and Zn onto porous IDEs. (j) High magnification SEM image of PANI on porous Au, again showing nanowire‐like morphology with notable porosity, promoting improved electrochemical interface. (k) Magnified SEM image of Zn electrodeposited on porous Au IDEs, displaying a similar mixed flake/nanosheet morphology.

Figure 3 presents SEM images of both flat and porous Au‐based Zn//I_2_ MBs at various magnifications, confirming the successful electrodeposition of PANI and Zn on the respective IDEs. SEM images of flat Au IDEs are shown in Figure S3. Figure 3a,b illustrates the overall electrodeposition of PANI and Zn on flat Au IDEs, while the high‐resolution image in Figure 3c highlights the nanowire‐like morphology and inherent porosity of the PANI cathode—features that can enhance electrolyte interaction and ion transport. Figure 3d depicts uniform Zn deposition with a mixed flake/nanosheet morphology on the flat Au IDE, indicating well‐controlled and homogeneous growth. Complementary SEM and EDS elemental mapping (Figure S4) further confirms the uniform distribution of carbon and nitrogen in the PANI cathode region, and Zn in the anode region. Figure 3e–g shows the SEM images of the 3D porous Au scaffold IDEs at different magnifications. Figure 3h–k displays SEM images of the porous Au Zn//I_2_ MB, demonstrating efficient electrodeposition of PANI and Zn onto the 3D porous Au scaffold. Low‐magnification images (Figure 3h,i) show complete and crack‐free coverage of the porous IDEs, indicating robust mechanical and structural integration. The high‐magnification image in Figure 3j reveals the preserved nanowire‐like and porous structure of the PANI cathode on the 3D framework. Similarly, Figure 3k illustrates a uniform Zn layer exhibiting a mixed flake/nanosheet morphology, conformally deposited without disrupting the scaffold architecture. Additional SEM and EDS mapping (Figure S5) confirm the consistent spatial distribution of C, N, and Zn within their respective electrode regions, validating the reliability and reproducibility of the fabrication strategy.

**FIGURE 4:**
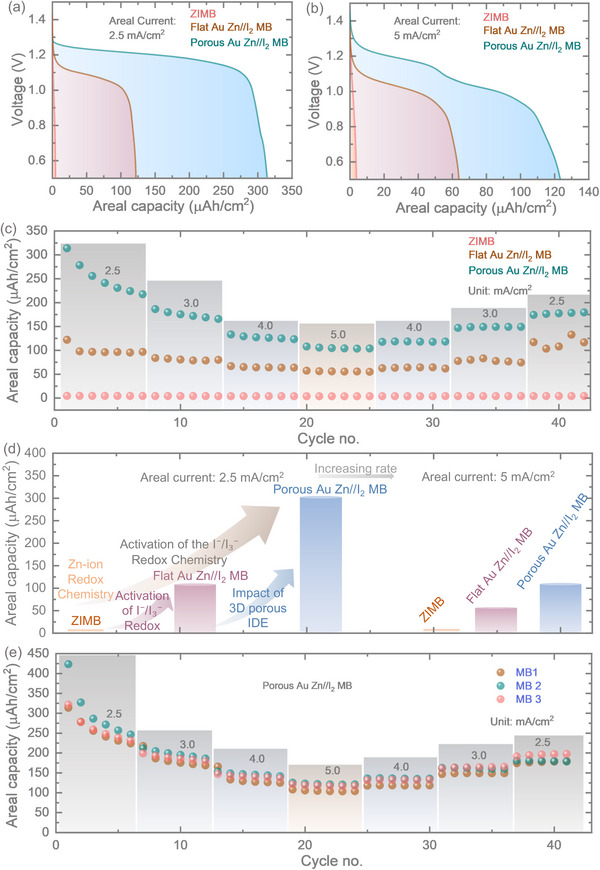
Galvanostatic discharge profiles of ZIMB, flat Au Zn//I_2_ MB, and porous Au Zn//I_2_ MB devices at areal currents of (a) 2.5 mA/cm^2^ and (b) 5.0 mA/cm^2^. (c) Rate performance comparison of the MBs across a range of areal currents, increasing from 2.5 mA/cm^2^ to 5.0 mA/cm^2^ and subsequently returning to 2.5 mA/cm^2^, demonstrating rate capability. (d) Comparative areal capacities of the MBs measured at 2.5 and 5 mA cm^−^^2^, illustrating that the activation of I^−^/I_3_^−^ redox chemistry markedly enhances charge‐storage performance within the same device footprint. The incorporation of 3D porous Au scaffolds further increases the capacity, underscoring the crucial role of electrode architecture. (e) Reproducibility assessment of porous Au Zn//I_2_ MBs (MB1, MB2, and MB3) under the same current regime, confirming consistent performance and reliable fabrication.

The charge storage performance of both flat Au Zn//I_2_ and porous Au Zn//I_2_ MB devices was evaluated by immersing them in a cuvette (Figure S6) filled with a gel electrolyte composed of 2 m Zn(CF_3_SO_3_)_2_ and 0.2 m ZnI_2_ in PVA (see Experimental Section for details). For baseline comparison, a ZIMB using flat Au IDEs with electrodeposited PANI and Zn was tested under the same conditions using a Zn(CF_3_SO_3_)_2_‐only electrolyte to isolate the Zn^2^^+^ storage contribution. Cyclic voltammetry (CV) curves of the ZIMB at various scan rates (1–2 mV/s) over 0.5–1.5 V (Figure S7a) exhibit two pairs of distinct redox peaks, attributed to the stepwise intercalation/deintercalation of Zn^2^^+^ into PANI [41]. Additional contributions from H^+^ doping/de‐doping during the redox process may also be present [42]. Increased scan rates lead to greater peak separation in the CV curves, indicative of higher overpotentials and slower reaction kinetics. The CV profiles of both flat and porous Au‐based Zn//I_2_ MBs (Figures S7b,c) exhibit well‐defined redox peaks around ∼1.17 V (reduction) and ∼1.30 V (oxidation), corresponding to the I^−^/I_3_^−^ redox couple vs Zn^2^^+^/Zn reference [28, 43]. During charging, Zn^2^^+^ ions gain electrons and are reduced to metallic Zn at the anode (Zn^2^^+^ + 2e^−^ → Zn), while I^−^ ions in the PANI polyelectrolyte lose electrons to form solid I_2_ (2I^−^ → I_2_ + 2e^−^), which subsequently reacts with excess I^−^ to form soluble I_3_^−^ species (I_2_ + I^−^ → I_3_^−^) (see further). Notably, the near overlap of CV curves at varying scan rates suggests minimal overpotential and fast redox kinetics, particularly for the porous Au architecture. Moreover, the Zn//I_2_ MBs exhibit significantly higher peak currents than the ZIMB, reflecting the fast and dominant I^−^/I_3_^−^ redox reactions. The sharp and well‐defined peaks further confirm rapid and reversible electrochemical processes. The galvanostatic charge‐discharge (GCD) test results for the ZIMB, flat Au Zn//I_2_ MB, and porous Au Zn//I_2_ MB devices at various areal current ranging from 2.5 to 5.0 mA/cm^2^ are given in Figures S8 and S9. The ZIMB delivers a low capacity of only 4.7 µAh/cm^2^ at 2.5 mA/cm^2^, whereas the flat Au Zn//I_2_ MB achieves 124 µAh/cm^2^ under the same conditions—exceeding a 26‐fold improvement. The porous Au Zn//I_2_ MB shows a substantial enhancement, reaching 311 µAh/cm^2^—representing a 151% improvement over the flat counterpart, enabled by the increased active material loading and improved conductivity of the 3D porous structure (Figure 4a). The porous Au Zn//I_2_ MB retains a high areal capacity of 122 µAh/cm^2^ even at 5.0 mA/cm^2^, demonstrating high‐rate performance (Figure 4b). Both flat and porous Au Zn//I_2_ MBs display stable discharge plateaus in the 2.5 – 5.0 mA/cm^2^ range, beneficial for high energy density operation. Rate performance plots (Figure 4c) further emphasize the superiority of the porous Au ZIMB across all areal currents, including during reverse current switching (5.0 → 2.5 mA/cm^2^), highlighting the structural stability and fast charge transport in the 3D scaffold. Specifically, the porous Au Zn//I_2_ MB achieves areal capacities of ∼187.25 and 137.49 µAh/cm^2^ at 3.0 and 4.0 mA/cm^2^, respectively. In comparison, the flat Au Zn//I_2_ MB delivers ∼ 87.35 and 70.30 µAh/cm^2^, while the ZIMB performs poorly (< 5 µAh/cm^2^) across the same current range. These results clearly demonstrate the superior charge storage capabilities of the porous Au Zn//I_2_ MB. The comparative areal capacities of the MBs measured at 2.5 and 5 mA cm^−^^2^ (Figure 4d) further illustrate that activation of the I^−^/I_3_^−^ redox chemistry significantly enhances charge‐storage performance within the same device footprint. Moreover, the incorporation of 3D porous Au scaffolds further boosts capacity, highlighting the critical role of electrode architecture. The reproducibility of the porous Au Zn//I_2_ MBs was validated by testing three independent devices (MB1, MB2, MB3), as shown in Figure 4e. All devices exhibited consistent rate performance during cycling between 2.5 and 5.0 mA/cm^2^, further reinforcing the reliability of the 3D electrode architecture and its potential for scalable, high‐performance MB applications. In addition, the Coulombic efficiency of the porous Au Zn//I_2_ MB was evaluated at different areal currents, progressively increasing from 2.5 to 5 mA cm^−^^2^, followed by a return to 2.5 mA cm^−^^2^, as shown in Figure S10. The device exhibits a Coulombic efficiency of approximately 99% at 5 mA cm^−^^2^, whereas a relatively lower Coulombic efficiency is observed at lower areal currents. This behavior indicates enhanced reversibility and faster charge storage kinetics at higher rates in the Zn//I_2_ MB system. Furthermore, for comparison, we investigated the electrochemical performance of AC loaded onto porous Au used for loading of PANI to serve as a micro‐cathode while Zn as an anode, and iodine host for Zn//I_2_ batteries (see Figure S11 shows the details of the characterizations of AC). The CV profiles of porous Au‐AC Zn//I_2_ MB (Figures S12a) exhibit well‐defined redox peaks corresponding to the I_3_^−^/I^−^ redox couple vs Zn^2^^+^/Zn reference [28]. The GCD profiles of the porous Au‐AC Zn//I_2_ MB at various areal currents (2.5–5.0 mA cm^−^^2^) are presented in Figure S12b. The device delivers areal capacities of 244.47, 144.34, 96.29, and 70.01 µAh cm^−^^2^ at 2.5, 3, 4, and 5 mA cm^−^^2^, respectively. Figure S12c summarizes the rate performance across the full current range (2.5 → 5.0 mA cm^−^^2^), including the recovery behavior during the reverse current sweep (5.0 → 2.5 mA cm^−^^2^). Long‐term cycling stability (Figure S12d) at 4 mA cm^−^^2^ shows that the capacity decreases from approximately 133 µAh cm^−^^2^ to 95 µAh cm^−^^2^ within the first ∼25 cycles, then remains relatively stable for nearly 150 additional cycles before further declining to 68 µAh cm^−^^2^ after 200 cycles. Although AC‐based materials typically exhibit stable capacities in Zn//I_2_ systems, this behavior is not observed in our AC‐loaded MBs. This discrepancy is likely related to the physical loading of AC within the confined porous Au IDE architecture, which may increase interfacial resistance, ultimately contributing to the observed capacity fading. In contrast, direct electrodeposition of PANI onto the porous Au IDEs effectively mitigates these limitations (see discussion below). As demonstrated through theoretical calculations, PANI exhibits strong adsorption affinity toward I^−^/I_3_^−^ species because of its electron‐donating nature. The presence of C─N bonds introduces chemically active nitrogen sites that engage in strong charge‐transfer interactions with iodine [44, 45]. Specifically, lone‐pair electrons on the imine (═N─) and amine (─NH─) groups donate electron density to I_2_, facilitating the formation of polyiodide species (I_3_^−^). The positively charged, protonated PANI matrix further stabilizes iodine through combined *π*–*π* interactions and its conjugated C─N─C backbone, enabling robust binding. In comparison, AC displays significantly weaker iodine adsorption, primarily governed by nonpolar C─C and C═C bonds that interact with iodine only through weak van der Waals forces. This results in physical rather than chemical adsorption, explaining the inferior iodine confinement and the poorer cycling stability observed in the porous Au‐AC Zn//I_2_ MB. The long‐term cycling Coulombic efficiency of the porous Au Zn//I_2_ MB device, measured at an areal current of 4 mA cm^−^^2^, is presented in Figure S13. The device exhibits a stable Coulombic efficiency of over 94.5% after 200 cycles. The Coulombic efficiency being slightly lower than 100% can be attributed to the incomplete reversibility of the Zn//I_2_ redox chemistry. Furthermore, the extended self‐discharge behavior of the porous Au Zn//I_2_ MB device is shown in Figure S14. The device maintains a voltage above 0.8 V for more than 15 h.

**FIGURE 5:**
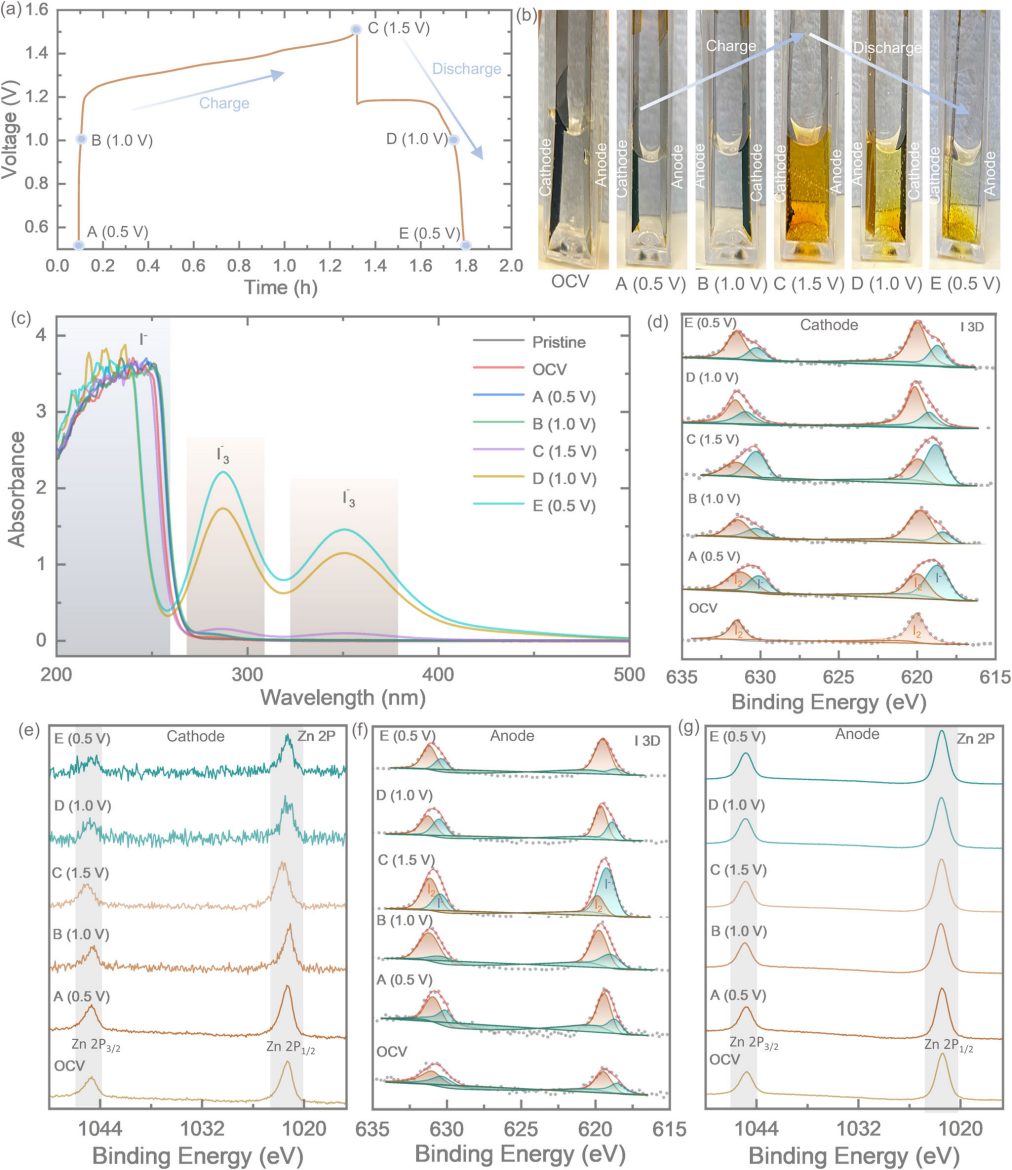
(a) GCD profiles of the Zn//I_2_ battery at different SoC, labeled A–E, using an electrodeposited PANI cathode on Ti foil and a Zn foil anode (first cycle). (b) Digital photographs of Zn//I_2_ battery cells at SoC points A–E, showing the electrodeposited PANI on Ti foil and Zn foil immersed in aqueous electrolyte. (c) UV–vis absorption spectra of the electrolytes corresponding to various SoCs levels, including the pristine state, open‐circuit voltage (OCV), and points A–E. (d) Deconvoluted I 3d XPS spectra of PANI cathodes at different SoC levels. (e) Zn 2p XPS spectra of PANI cathodes at various SoC levels. (f,g) Deconvoluted I 3d and Zn 2p XPS spectra of Zn anodes measured at different SoC states.

To gain deeper insight into the charge storage mechanism of Zn//I_2_ batteries, we performed ex situ analyses at various state‐of‐charge (SoC) levels, as illustrated in Figure 5. Corresponding electrodes were examined using XPS and SEM, while the electrolytes at different SoCs were characterized via UV–vis spectroscopy. For the SoC‐dependent measurements, a PANI cathode electrodeposited on Ti foil was paired with a Zn foil anode. Both electrodes were immersed in a cuvette containing an aqueous electrolyte composed of 2 m Zn(CF_3_SO_3_)_2_ and 0.2 m ZnI_2_. The cells were charged or discharged and held at specific voltages corresponding to SoC points A–E, as marked in Figure 5a. Digital images of the cells at these different SoCs (Figure 5b) show a clear color change in the electrolyte—from transparent to light yellow—indicating the electrochemical conversion of I^−^ to I_3_^−^ [46]. The UV–vis spectra in Figure 5c confirm the presence of I^−^ at all states, including the pristine electrode, open‐circuit voltage (OCV), and SoC points A (0.5 V), B (1.0 V), C (1.5 V), D (1.0 V), and E (0.5 V). Notably, as the cell is charged from A (0.5 V) to higher potentials (e.g., C at 1.5 V), characteristic I_3_^−^ peaks emerge around ∼286 and ∼350 nm [46]. This spectral evolution corresponds to the oxidation of I^−^ ions within the PANI polyelectrolyte into I_2_, which subsequently reacts with residual I^−^ to form soluble I_3_^−^, manifesting as the yellow coloration of the electrolyte [47]. To further understand this redox behavior, XPS analysis was conducted on the electrodes at various SoCs (Figure 5d–g). Figure 5d presents the I 3d spectra of the PANI cathode. At OCV (1.1 V), characteristic peaks for I 3d_5_/_2_ and I 3d_3_/_2_ appear at ∼ 631.53 and ∼ 619.97 eV, respectively. Upon full discharge at point A (0.5 V), the I 3d peaks shift to lower binding energies, with deconvoluted peaks at ∼ 631.31 and ∼ 630.13 eV (I 3d_5_/_2_) and at ∼ 619.97 and ∼ 618.80 eV (I 3d_3_/_2_), indicating partial reduction of I_2_ to I^−^ and the coexistence of both oxidation states [48, 49]. At the fully charged state (point C, 1.5 V), the spectra show a dominant I^−^ peak (∼ 630.36 eV) alongside a smaller I_2_ peak (∼ 631.55 eV), confirming oxidation of I^−^ to I_2_. Upon returning to the fully discharged state at point E (0.5 V), the I 3d peaks shift back to higher binding energies, with a dominant I_2_ peak at ∼ 631.50 eV and a minor I^−^ peak at ∼ 630.30 eV, suggesting substantial conversion of I^−^ back to solid I_2_ [47, 50]. These results confirm the dynamic coexistence and interconversion of I^−^ and I_2_ species during the charge‐discharge process. This redox mechanism involves I^−^ ions in the PANI matrix losing electrons during charging to form solid I_2_ (2I^−^ → I_2_ + 2e^−^), which subsequently dissolves into I_3_^−^ via the reaction I_2_ + I^−^ → I_3_^−^ [28]. Ideally, the cathode is expected to be I^−^‐dominant in the discharged state and I_2_/I_3_^−^‐dominant in the charged state. However, it is important to note that the XPS measurements were performed at different states of charge during the initial cycle, where incomplete reversibility and kinetic limitations may influence the observed species distribution. Additionally, the Zn 2p XPS spectra for the Zn anode at different SoC states (Figure 5e) shows consistent signals for both Zn 2p_1/2_ (∼1044 eV) and Zn 2p_3/2_ (∼1021 eV), confirming the presence of Zn across all charge states (A–E). Similar XPS trends for both I 3d and Zn 2p are also observed for the Zn anodes (Figure 5f,g). Notably, the detection of iodine signals on the Zn anode surface suggests iodide embedded, which may contribute to side reaction effects on the Zn surface (see further discussion).

To further investigate structural and compositional changes during cycling, SEM imaging and EDS mapping were performed on the electrodes at various states of charge (Figures S15–S18). The SEM images of the PANI cathodes show that the characteristic nanowire morphology of PANI is largely retained throughout the cycle. However, some surface cracks are observed, which may result from the direct electrodeposition of PANI onto the Ti substrate without binders or conductive additives. EDS mapping of the PANI cathode at SoC point E (0.5 V) (Figure S15f) confirms the presence of C, I, and Zn elements. Similarly, SEM images and EDS mapping of the PANI cathode at various SoC points starting from OCV, A (0.5 V) to D (1.0 V) (Figures S16–S18) confirm the presence of C, I, and Zn elements.

**FIGURE 6:**
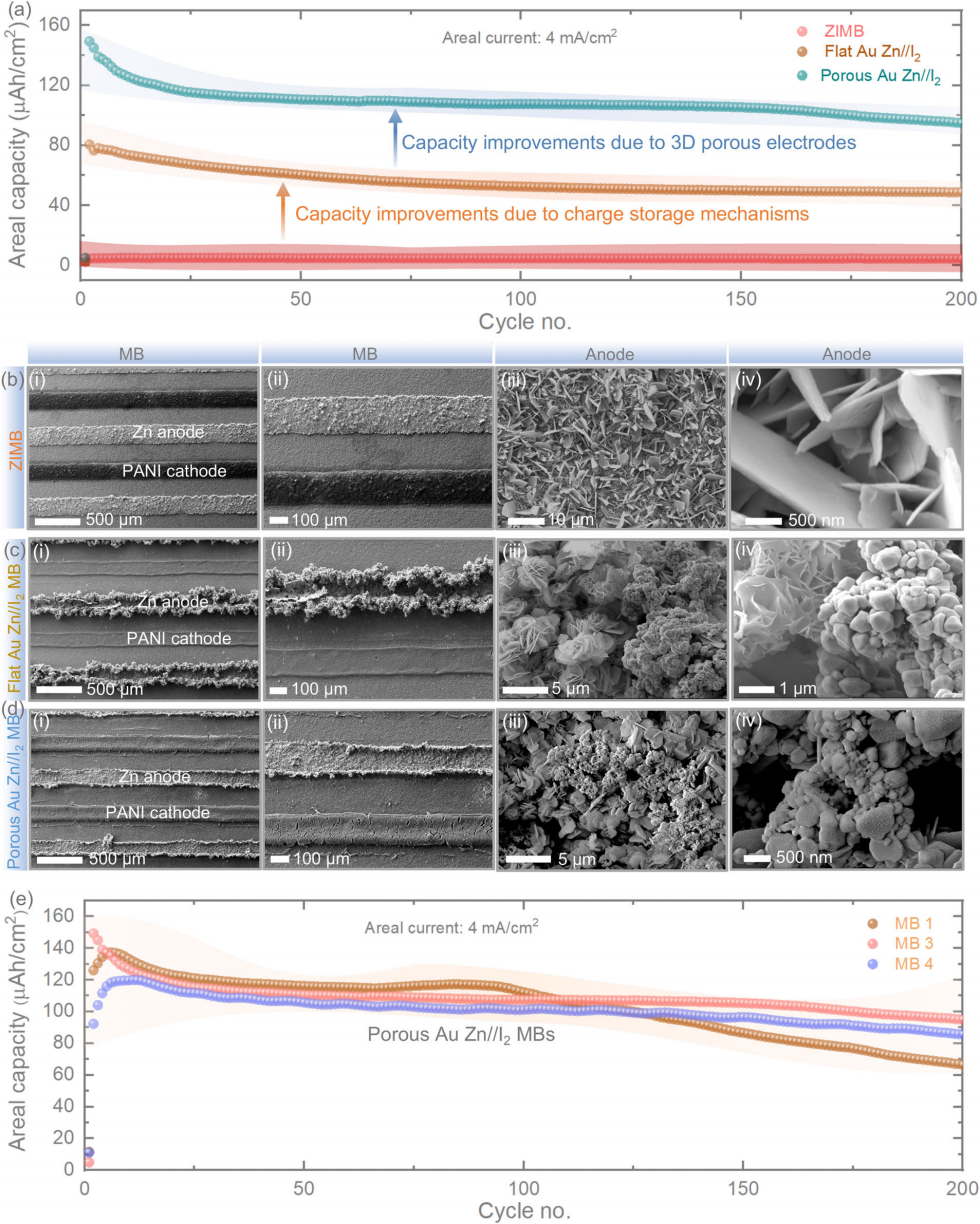
(a) Long‐term cycling performance comparison of ZIMB, flat Au Zn//I_2_ MB, and porous Au Zn//I_2_ MB devices over 200 cycles at a fixed areal current density of 4 mA cm^−^^2^. (b, i–ii) Post‐cycling SEM images of ZIMB at low magnification show intact adhesion of the PANI cathode and Zn anode to the flat Au electrode, with no signs of delamination. (b, iii–iv) High‐magnification SEM images of the Zn anode reveal flake‐like Zn deposits formed after extended cycling. (c, i–ii) SEM images of the flat Au Zn//I_2_ MB after cycling show secure electrode adhesion, but significant morphological changes on the Zn anode due to dendrites and polyiodide‐induced corrosion. (c, iii–iv) High‐magnification images show flake‐like Zn dendrites and discrete polyiodide chain structures. (d, i–ii) SEM images of the porous Au Zn//I_2_ MB demonstrate stable electrode adhesion and reduced morphological changes on the Zn anode compared to the flat counterpart. (d, iii–iv) High‐magnification images reveal reduced flake‐like Zn deposits and fewer polyiodide chain features, attributed to the beneficial effect of the 3D porous Au architecture. (e) Reproducibility test of three porous Au Zn//I_2_ MB devices (MB1, MB2, MB3) at 4 mA cm^−^^2^ shows consistent cycling performance, highlighting the robustness and reliability of the porous electrode design.

To evaluate the long‐term cycling stability of MBs, GCD cycling tests were conducted on ZIMB, flat Au Zn//I_2_ MB, and porous Au Zn//I_2_ MB devices. As shown in Figure 6a, all devices exhibit stable cycling performance up to 200 cycles. Consistent with the CV and GCD results, the porous Au Zn//I_2_ MB demonstrates reversible charge storage performance, maintaining the stable areal capacity after 200 cycles. Specifically, the areal capacities at 4 mA cm^−^^2^ were 96 µAh cm^−^^2^ for the porous Au Zn//I_2_ MB, 49 µAh cm^−^^2^ for the flat Au Zn//I_2_ MB, and 5 µAh cm^−^^2^ for the ZIMB, while Figure S19 shows the digital photographs of the flat and porous Au Zn//I_2_ MB devices after 200 cycles. To further investigate microelectrode stability, post‐cycling SEM imaging and Raman spectroscopy were performed. The ex situ Raman analysis of the PANI cathodes before and after cycling for Zn//I_2_ MBs is illustrated in Figure S20. The characteristic Raman peaks of cycled PANI cathodes are identical to those detected before cycling, demonstrating that PANI stability is maintained even after prolonged cycling. Figure 6b–d presents post‐cycling SEM images of ZIMB, flat Au Zn//I_2_ MB, and porous Au Zn//I_2_ MB microelectrodes at various magnifications. Figure 6b shows postmortem SEM images of the ZIMB device. Low‐magnification SEM images (i, ii) indicate no obvious electrode detachment, dendrite overgrowth, or severe side reactions on the Zn micro anode. High‐magnification SEM images (iii, iv) reveal flake‐like Zn deposits on the Zn anode and minor cracks in the PANI cathode (Figure S21a–c), while the porous nanowire structure of the PANI remains largely intact. EDS mapping of the ZIMB electrodes (Figure S21d–i) confirms the uniform distribution of Zn on the anode and C, N on the PANI cathode. The flat Au Zn//I_2_ MB (Figure 6c) shows significant morphological changes in the Zn anode after 200 cycles. Low‐magnification SEM images (i, ii) reveal volume expansion and surface degradation, likely due to Zn dendrite formation and polyiodide‐induced side reaction [51]. High‐resolution SEM images (iii, iv) show extensive growth of flake‐like Zn deposition and polyiodide chain clusters forming secondary microparticles. In contrast, the PANI cathode (Figure S22a–c) retains its nanowire‐like morphology with only minor cracking (Figure S22b). For the porous Au Zn//I_2_ MB, SEM images (Figure 6d) show minimal morphological changes in the Zn anode, which is attributed to the protective effect of the 3D porous Au current collector. High‐resolution SEM images reveal only few particles formation due to polyiodide interaction [51]. The PANI cathode (Figure S23a–c) maintains its nanowire structure with distinct porosity but exhibits cracks due to mechanical stress from high mass loading and cycling. EDS mappings (Figures S22 and S23) confirm the presence of key elements (Zn at the anode and C, N at the PANI cathode) for both flat and porous architectures after extended cycling. Finally, the reproducibility of the cycling performance was demonstrated by testing three porous Au Zn//I_2_ MBs—designated MB1, MB2, and MB3 – under the same areal current (4 mA cm^−^^2^). As shown in Figure 6e, all three devices consistently outperformed the flat Au Zn//I_2_ MB and ZIMB, highlighting the effectiveness and reproducibility of the 3D porous electrode design in enhancing the charge storage performance of Zn//I_2_ MBs.

To evaluate the MB performance under reduced electrolyte volume and more practical conditions, a compact packaging configuration was developed using transparent double‐sided tape and Kapton tape (Figure S24a). Two porous Au PANI//Zn MBs were assembled: one configured as a Zn//I_2_ MB using 120 µL of 2 m Zn(CF_3_SO_3_)_2_ + 0.2 m ZnI_2_ PVA gel electrolyte, and the other as a ZIMB using 120 µL of 2 m Zn(CF_3_SO_3_)_2_ PVA gel electrolyte. Both devices were evaluated under conditions at an areal current of 2.5 mA cm^−^^2^. The Zn//I_2_ MB delivers an initial areal capacity of 188 µAh cm^−^^2^ compared to 28 µAh cm^−^^2^ for the ZIMB, reflecting the additional iodine redox contribution. After 50 cycles, the Zn//I_2_ MB retains an areal capacity of 145.70 µAh cm^−^^2^, while the ZIMB maintains 28 µAh cm^−^^2^, with both systems exhibiting coulombic efficiencies exceeding 95%. These results demonstrate the feasibility of translating the device into a compact configuration compatible with on‐chip and wearable applications while maintaining high electrochemical performance. Furthermore, the porous Au Zn//I_2_ MBs functionality was demonstrated by its effective operation in powering commercial sensors. Two fully charged porous Au Zn//I_2_ MB connected in series powered an indoor‐outdoor thermometer with a hygrometer clock, as shown in Figure S25, highlighting the potential for broader applications of on‐chip MBs. We evaluated the areal energy and power densities of our porous Au Zn//I_2_ MB and flat Au Zn//I_2_ MB in comparison with previously reported MBs, as illustrated in the Ragone plot (Figures S26 and S27). The porous Au Zn//I_2_ MB achieves a peak areal energy of 363.36 µWh cm^−^^2^ and a peak areal power of 5385 µW cm^−^^2^. These results underscore the advantages of our engineered microelectrode architecture and the Zn//I_2_ redox system in achieving high electrochemical performance within a miniaturized footprint. Overall, this work demonstrates a promising path toward the development of high‐performance, on‐chip energy storage systems, which are vital for powering next‐generation smart system‐on‐chip technologies.

## 3 Conclusions

This study demonstrates a significant advancement in the development of safer, high‐performance on‐chip Zn‐based MB systems by introducing ZnI_2_ as a dual ion electrolyte to activate the I^−^/I_3_^−^ redox chemistry on conventional Zn anodes and PANI cathodes. This approach leads to an enhancement in charge storage performance—exceeding a 26‐fold improvement compared to traditional Zn‐ion storage mechanisms. Further enhancement is achieved by combining this with a 3D porous current collector, which, combined with electrodeposited PANI, a Zn anode, and the ZnI_2_‐modified electrolyte, delivers a ∼151% improvement in areal capacity over flat Au‐based devices. The 3D porous Au framework enables efficient, high‐mass loading of active materials within a limited footprint via electrodeposition. The resulting porous Au Zn//I_2_ MB achieves an areal capacity of 313.73 µAh cm^−^^2^ at 2.5 mA cm^−^^2^, along with improved areal energy and power of 363.25 and 5385 µW cm^−^^2^.

## Conflicts of Interest

The authors declare no conflicts of interest.

## Supporting information




**Supporting File**: advs75453‐sup‐0001‐SuppMat.pdf.

## Data Availability

The data that support the findings of this study are available from the corresponding author upon reasonable request.
