# Supplementary material for: 3D Engineered Dual‐Redox Zinc‐Iodine Microbatteries for Intrinsically Safe on‐Chip Energy Storage
Source: Adv Sci (Weinh). 2026 Apr 27;13(41):e75453. doi: 10.1002/advs.75453 (PMC13335492; doi:10.1002/advs.75453)
Supplement: Supplementary file 1 — Supporting File: advs75453‐sup‐0001‐SuppMat.pdf. [file ADVS-13-e75453-s001.pdf]

Supporting Information

for

3D Engineered Dual-Redox Zinc-Iodine Microbatteries for Intrinsically Safe On-Chip Energy Storage

Nibagani Naresh,[1] Sanat Nalini Paltasingh,[2] Yijia Zhu,[1] Xiaopeng Liu,[1] Yujia Fan,[1] Monojit Mondal,[1] Su Jin Heo,[3] Shuhui Li,[4] Mingqing Wang,[1] Shaoliang Guan,[5] Yanzhuo Li,[1] Georgios Nikiforidis,[1] Shahab Akhavan,[1] Ivan P. Parkin,[4] Saroj Kumar Nayak,[2] Michael De Volder,[3] Buddha Deka Boruah[*,1]

[1]Institute for Materials Discovery, University College London, London WC1E 7JE, United Kingdom

[2]School of Basic Sciences, Indian Institute of Technology Bhubaneswar, Khorda, Odisha 752050, India

[3]Institute for Manufacturing, Department of Engineering, University of Cambridge, Cambridge, CB3 0FS, UK

[4]Department of Chemistry, University College London, London, WC1H 0AJ, UK

[5]Department of Materials Science & Metallurgy, University of Cambridge, Cambridge, CB3 0FS, UK

*Corresponding author: Dr. Buddha Deka Boruah

Email: b.boruah@ucl.ac.uk

Experimental Section

Materials: The electrodeposition of 3D porous Au was carried out using gold (III) chloride trihydrate ($AuCl_4H_7O_3$) and ammonium chloride (NH_4Cl), both procured from Sigma-Aldrich. For the electrodeposition of PANI, sulfuric acid (H_2SO_4) and aniline, both sourced from Sigma-Aldrich, were used. Sodium sulfate (Na_2SO_4), zinc sulfate heptahydrate ($ZnSO_4 \cdot 7H_2O$), and boric acid (H_3BO_3), all obtained from Sigma-Aldrich, were utilized for the electrodeposition of Zn. The gel electrolyte prepared using Zn $(CF_3SO_3)_2$, Zinc Iodide (ZnI_2) and, Polyvinyl alcohol (PVA) both obtained from Sigma-Aldrich. The plane Au IDEs (Interdigitated Gold Electrodes with 200-micron lines and gaps, DRP-IDEAU200) were obtained from Metrohm U.K. Ltd. All chemicals were used without further purification.

Electrodeposition of porous Au scaffold: The Au IDE (Interdigitated Gold Electrodes with 200-micron lines and gaps, DRP-IDEAU200) devices were cleaned with isopropanol and distilled water, followed by drying with compressed air. A solution of 0.1 M gold (III) chloride trihydrate ($AuCl_4H_7O_3$) and 2 M ammonium chloride (NH_4Cl) was prepared in 20 mL of distilled water to serve as the electrolyte for the electrodeposition of porous Au using the dynamic bubble technique. Ag/AgCl and Platinum (Pt) electrode were used as reference electrode and counter electrode. The porous Au electrodeposited at a voltage of -2.1 V for 6 S. After electrodeposition the deposited Au IDEs were rinsed with distilled water and allowed to dry. The electrodeposition was carried out using an Autolab electrochemical workstation.

Electrodeposition of PANI: The PANI electrolyte solution was prepared by mixing 1 M H_2SO_4 (95-97%) and 0.5 M aniline in 50 ml of distilled water and stirred continuously for 3 h. Platinum (Pt) and Ag/AgCl electrode were used as counter electrode and reference electrode. The PANI electrodeposited on plane Au IDEs and 3D porous Au IDEs by electrodeposition. The deposition was performed at constant potential of 0.85 V for 30 s on plane Au IDEs and 3D porous Au IDE devices. After electrodeposition the electrodes were washed with distilled water and allowed to dry.

Electrodeposition of Zinc: Zn was electrodeposited on one side of both porous Au IDE devices and flat Au IDE devices using an electrodeposition technique. The Zn electrolyte solution was prepared by dissolving 22.3 g of zinc sulfate heptahydrate ($ZnSO_4 \cdot 7H_2O$), 12.5 g of sodium sulfate (Na_2SO_4) and 2 g of boric acid (H_3BO_3) in 91 mL of deionized water. The electrodeposition process was performed at a current of -40 mA for 10 seconds on both planar

Au IDEs and 3D porous Au IDEs. Following electrodeposition, the IDE devices were rinsed with distilled water and left to dry.

Preparation of electrode inks: Electrode inks were made by mixing 10 wt% PVDF in dimethylformamide (DMF) as a binder and Super-P carbon black as a conductive additive. The composition was adjusted to have an active substance, conductive additive, and binder weight ratio of 88:10:2. Specifically, 0.88 g of AC powder, 0.1 g of Super-P carbon black, and 0.2 g of 10 wt% PVDF in DMF solution were utilized. Further, 5.5 g of TEGMME was incorporated into the AC electrode ink. A Fisherbrand 505 sonicator was used to sonicate each mixture for 30 minutes using the tip sonication. The sonicator's amplitude was set to 30%, with a pulse configuration of 0101. After sonication, electrode inks were homogeneous.

Printing of devices: SonoPlot Microplotter Proto with 20 μm nozzle was used to print the pattern drawn with SonoDraw software. After electrodeposition of porous Au on plane Au IDEs, the nozzle was aligned with the starting point of the IDE pattern. The feature size and printing mode were set to 50 μm and spraying mode, respectively, with a printing voltage of 12 V for AC electrode ink.

Preparation of gel electrolyte for micro-batteries: Clear transparent gel electrolyte was prepared by dissolving 1 g of PVA in 10 g of distilled water in oil bath at 85 ^{o}C for one hour to obtain uniform transparent gel. After that, 2M Zn $(CF_3SO_3)_2$ and 0.2 M Zinc Iodide (ZnI_2) powder was dissolved in above gel in oil bath for 2 h to get uniform transparent gel electrolyte.

Materials characterization: A scanning electron microscope (SEM) (Zeiss EVO LS15) was used to investigate the morphology of the devices. Additionally, a stylus profilometer (Bruker DektakXT) was used to examine the devices' surface profiles. XRD measurements of PANI and Zn were performed on an AERIS PANalytical Research Edition machine using Cu Kα radiation (λ = 0.154 nm) at 40 kV and 7.5 mA. The scan rate was 5° per minute from 10° to 80°. The Raman spectra of PANI were measured using a Renishaw inViaTM confocal Raman microscope with a 532 nm laser, which spans the 100–3200 cm^{-1} range.

Electrochemical tests of micro batteries: Both ends of the Au IDEs was connected with Cu foil using silver paste and immersed vertically in cuvette (Fisherbrand Disposable Cuvettes 14955125) filled with gel electrolyte. Both ends of the Cu foil are well separated and properly packed using kepton tape to avoid short circuit and top of the cuvette sealed with parafilm. The volume of the electrolyte used is 1 ml for all Zn//I_2 MBs. Galvanostatic discharge-charge (GDC) was measured at areal currents from 50 to 1000 $\mu A/cm^2$, and cyclic voltammetry (CV)

was measured at scan rates ranging from 0.3 mV/s over a voltage window of 0.5 to 1.5 V for micro-batteries using an electrochemical instrument (Biologic). A Neware battery tester was used for long-term cycle measurements. An Autolab electrochemical workstation was used to perform electrochemical impedance spectroscopy (EIS) measurements. The frequency testing range was 10 mHz to 100 kHz at OCP with a voltage amplitude of 10 mV.

Computational details: First-principles calculations were carried out using Density Functional Theory (DFT),[1–3] using the Vienna Ab Initio Simulation Package (VASP),[4,5] code for theoretical investigation. The generalized gradient approximation[6] of Perdew-Burke-Ernzerhof[7] (GGA-PBE) was employed to characterize the exchange correlation potential. A plane-wave cutoff energy of 500 eV was considered for all calculations. All geometrical structures were optimized by fully relaxing atomic positions until the Hellman-Feynman forces on each atom were reduced below a threshold of 0.01 eV, with a total energy convergence criterion of 1×10^{-5} eV. To include van der Waals interactions, the DFT-D3 method[8] was applied to correct for potential energy and interatomic forces. For structure optimization, the Brillouin zone was sampled using gamma-centred k-point grids of 3×3×1.

The binding energies of the adsorbed molecules or atoms on each surface were calculated using the following formula:

$$\mathrm{E_b} = \mathrm{E_{surface+molecule/atom}} - \mathrm{E_{surface}} - \mathrm{E_{molecule/atom}}$$

Where, $\mathrm{E_{surface+molecule/atom}}$, $\mathrm{E_{surface}}$, and $\mathrm{E_{molecule/atom}}$ represent the total energies of the system with the molecule or atom adsorbed on the surface, the clean surface, and the isolated molecule or atom, respectively. According to this definition, a more negative binding energy value indicates a stronger interaction between the adsorbate and the surface.

The charge density difference (CDD) was calculated using the following expression:

$$\Delta\rho = \rho_{surface+molecule/atom} - \rho_{surface} - \rho_{molecule/atom}$$

Where, $\rho_{surface+molecule/atom}$ denotes the electron charge density of the surface with the adsorbed molecule or atom, $\rho_{surface}$, while $\rho_{molecule/atom}$ represent the electron charge densities of the clean surface and the isolated molecule or atom, respectively.

The work function (F) represents the minimum energy required for an electron to escape from the surface into the vacuum and is calculated using the following expression:

$$F = E_{vacuum} - E_{Fermi}$$

TABLE S1: *Bader charge analysis showing the charge transfer from the host surface to the I_3^-/I^-.*

	Polyaniline	Coronene	Graphite	Porous Graphite
I_3^-	0.45 |e|	0.34 |e|	0.42 |e|	0.35 |e|
I^-	0.42 |e|	0.32 |e|	0.39 |e|	0.35 |e|

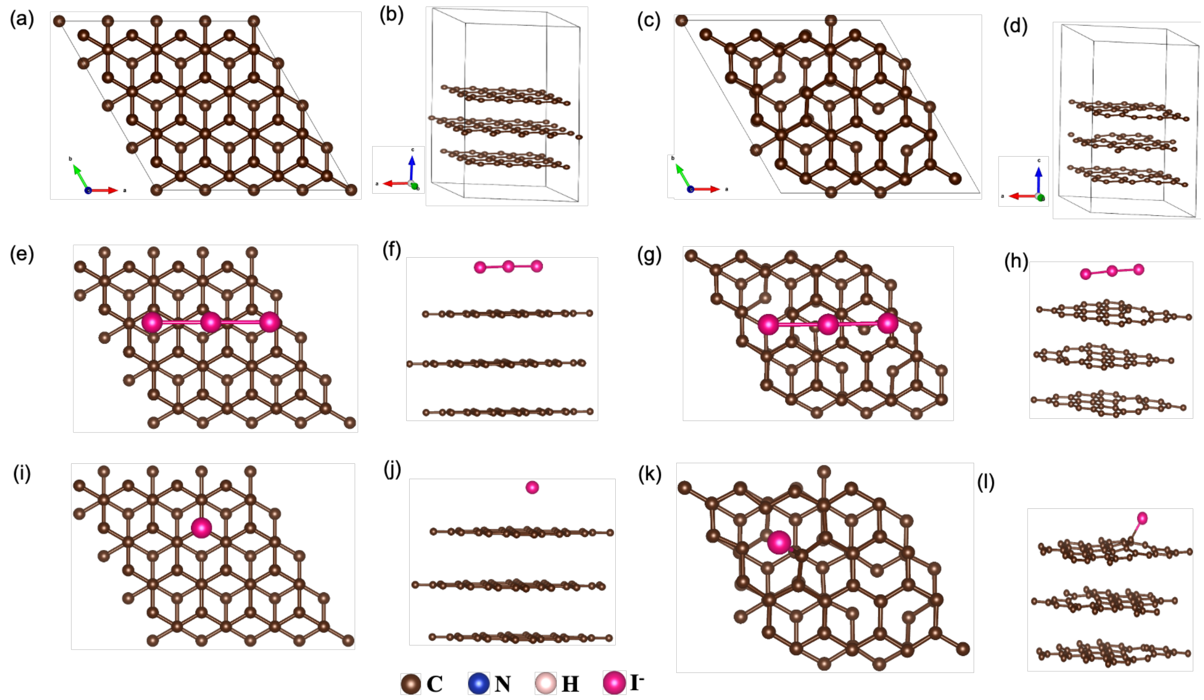

Figure S1. *Optimized structures of graphite (a)Top view, (b) Side view, and porous graphite (c) Top view, (d) Side view, respectively. Optimised structure of I_3 adsorbed on graphite (e) Top view, (f) Side view, and on porous graphite (g) Top view, (h) Side view, respectively. Optimised structure of I adsorbed on graphite (i) Top view, (j) Side view, and on porous graphite (k) Top view, (l) Side view, respectively. The dark grey, blue, off-white, and pink spheres represent carbon, nitrogen, hydrogen and iodine atoms, respectively.*

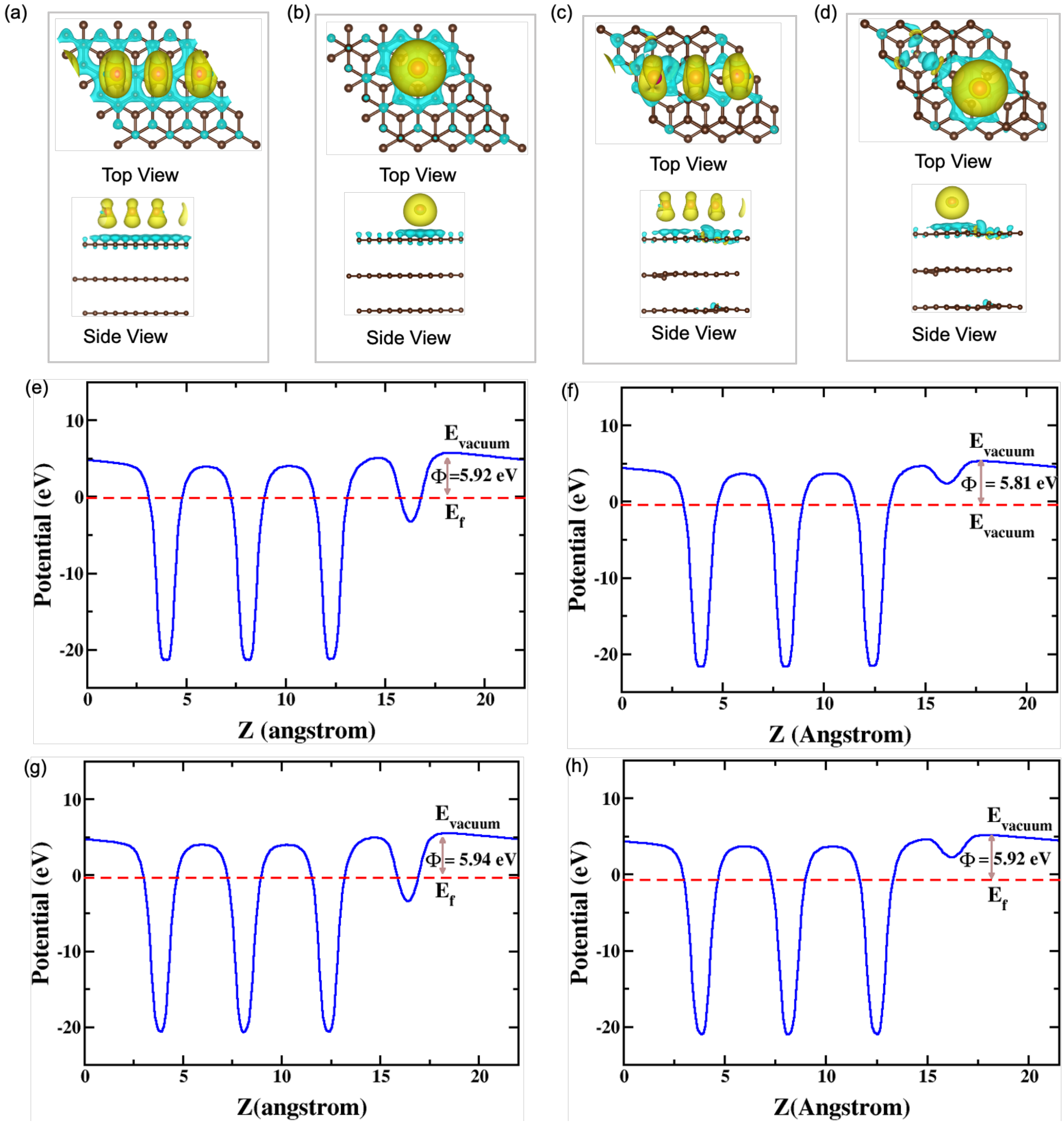

Figure S2. *Charge density difference (CDD) plot for (a) I_3^- adsorbed on graphite, (b) I^- adsorbed on graphite, (c) I_3^- adsorbed on porous graphite, and (d) I^- adsorbed on porous graphite. The yellow color represents the charge accumulation region, while the cyan color indicates the charge depletion region, with an iso-surface value of 0.0007 e/Å³. Workfunction plot of (e) I_3^- adsorbed on graphite, (f) I^- adsorbed on graphite, (g) I_3^- adsorbed on porous graphite, and (h) I^- adsorbed on porous graphite.*

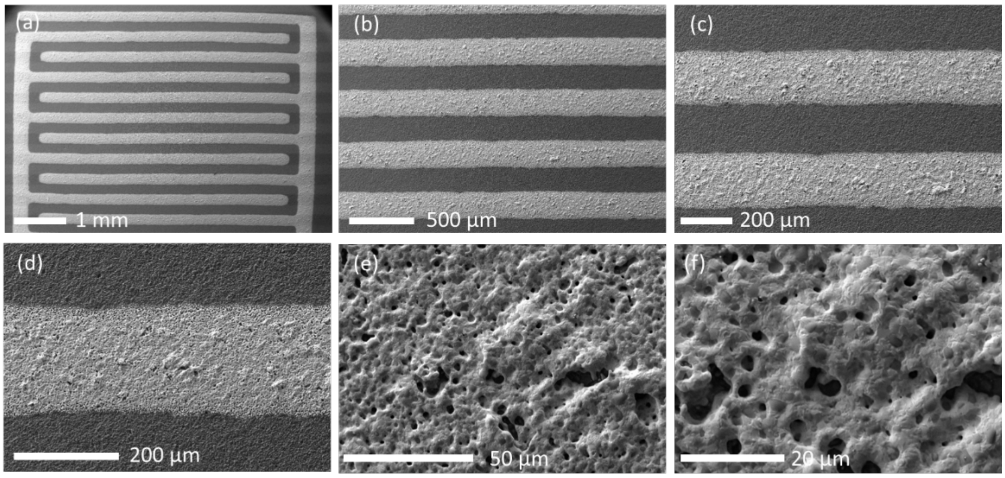

Figure S3. *(a-f) SEM images of flat Au IDEs at various magnifications.*

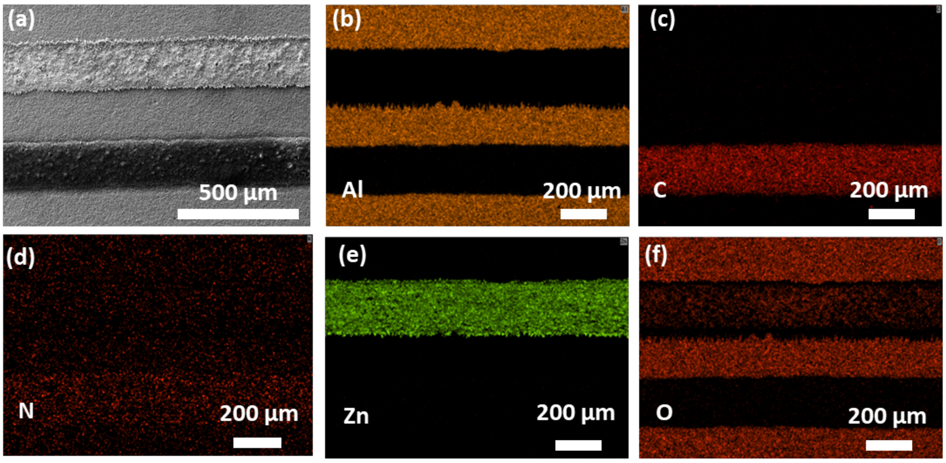

Figure S4. *(a) SEM images of flat Au Zn//I_2 MB at low magnification show intact adhesion of the PANI cathode and Zn anode to the flat Au electrode. (c, d) EDS elemental mapping of the PANI cathode shows the distribution of C, and N elements. (e) EDS elemental mapping of the Zn anode shows the distribution of Zn element and (b, f) EDS elemental mapping of the ceramic substrate shows distribution of Al and O elements.*

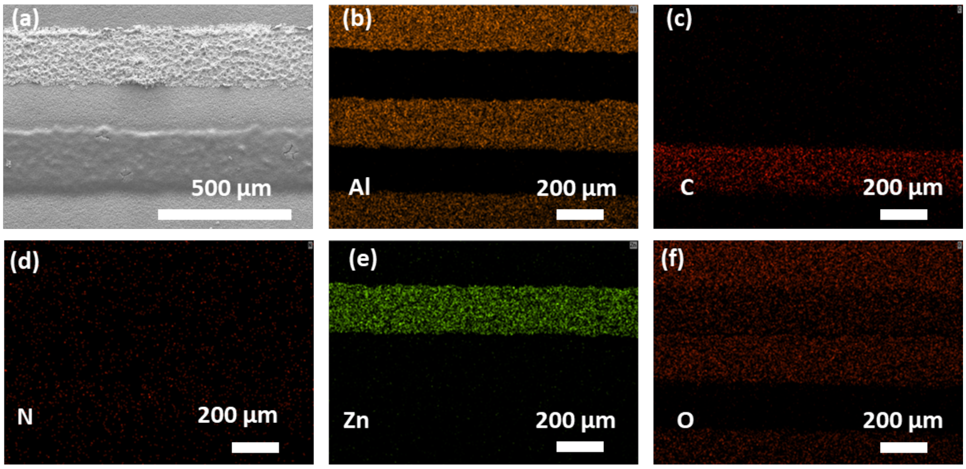

Figure S5. *(a) SEM images of porous Au Zn//I_2 MB at low magnification show intact adhesion of the PANI cathode and Zn anode to the flat Au electrode. (c, d) EDS elemental mapping of the PANI cathode shows the distribution of C, and N elements. (e) EDS elemental mapping of the Zn anode shows the distribution of Zn element and (b, f) EDS elemental mapping of the ceramic substrate shows distribution of Al and O elements.*

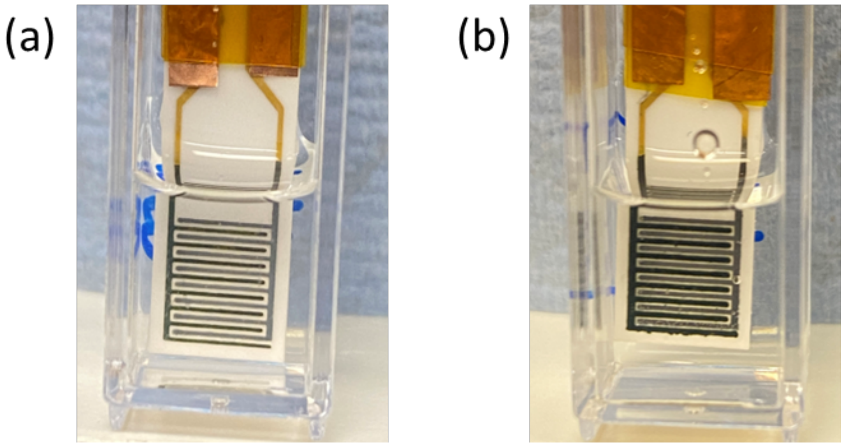

Figure S6. *Digital images of flat Au Zn//I_2 MB and porous Au Zn//I_2 MB devices before cycles vertically immersed into a cavate filled with 2M $Zn(CF_3SO_3)_2$ and 0.2 M ZnI_2 PVA transparent gel electrolyte.*

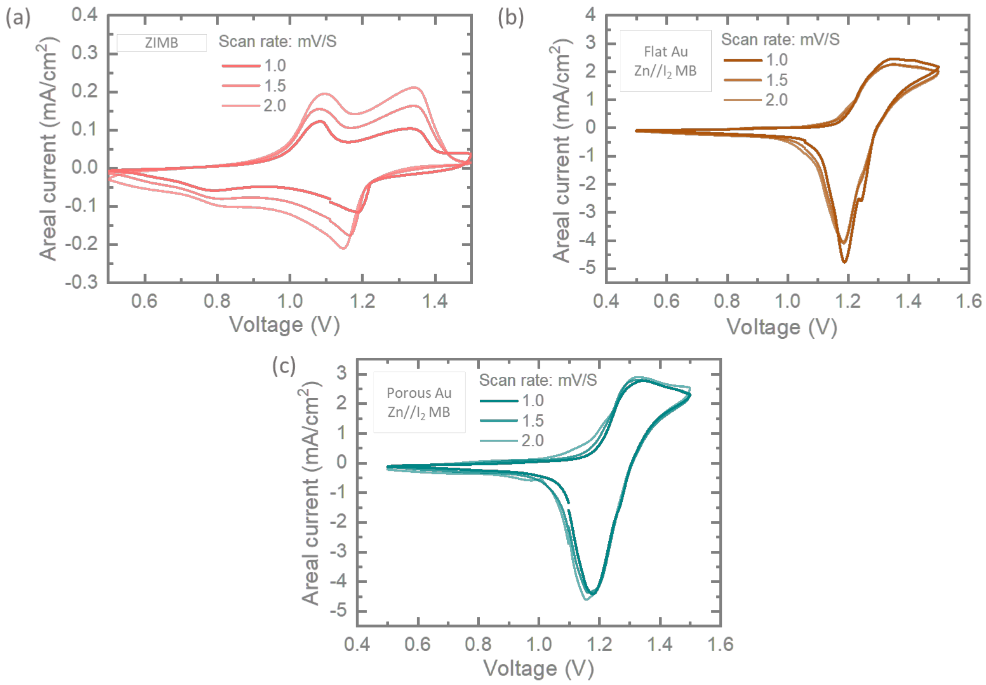

Figure S7. *Cyclic voltammogram curves of ZIMB, flat Au Zn//I_2 MB and porous Au Zn//I_2 MB devices at scan rate of 1 mV/s, 1.5 mV/s and 2 mV/s.*

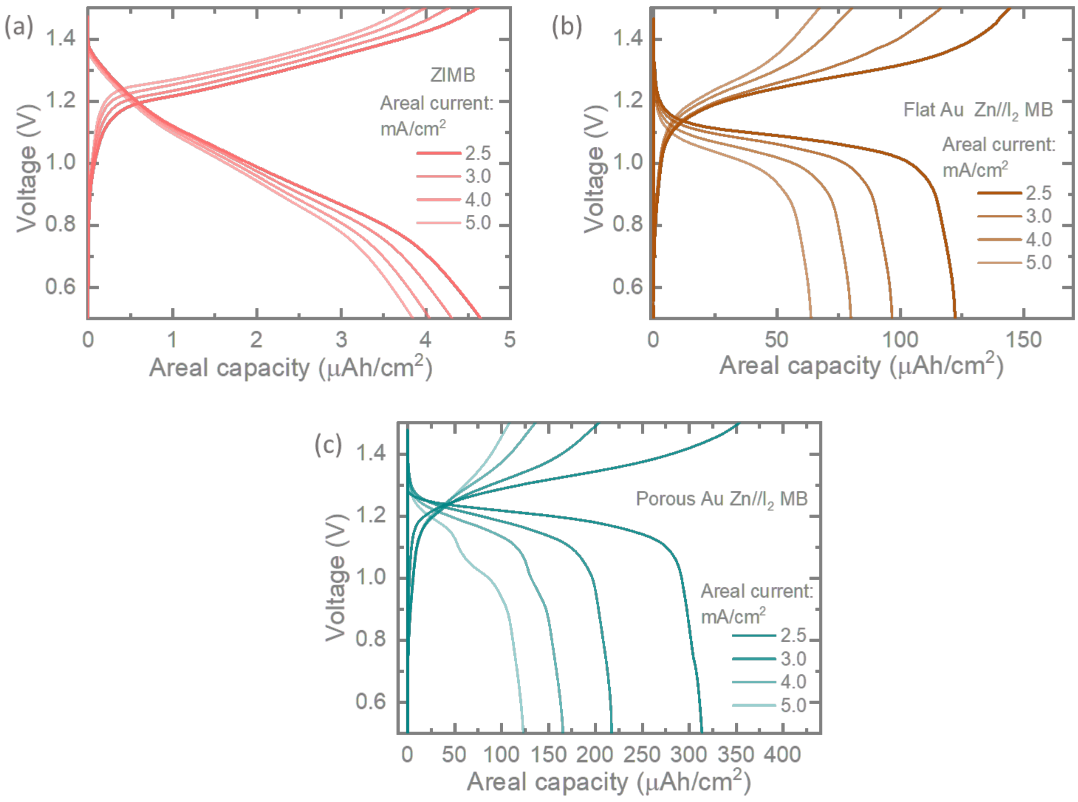

Figure S8. *GCD curves of ZIMB, flat Au Zn//I_2 MB and porous Au Zn//I_2 MB devices at varying areal current of 2.5 to 5 mA/cm^2.*

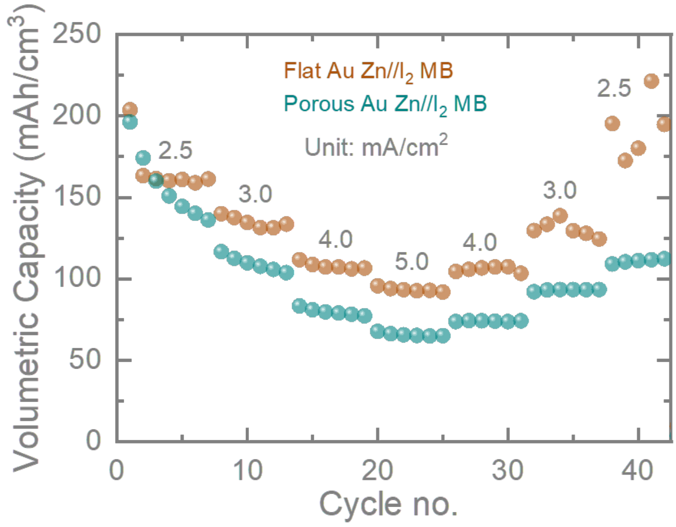

Figure S9. *Comparison of volumetric capacity rate test of flat Au Zn//I_2 MB and porous Au Zn//I_2 MB devices across different areal currents, progressively increasing from 2.5 mA/cm² to 5 mA/cm², followed by a return to 2.5 mA/cm².*

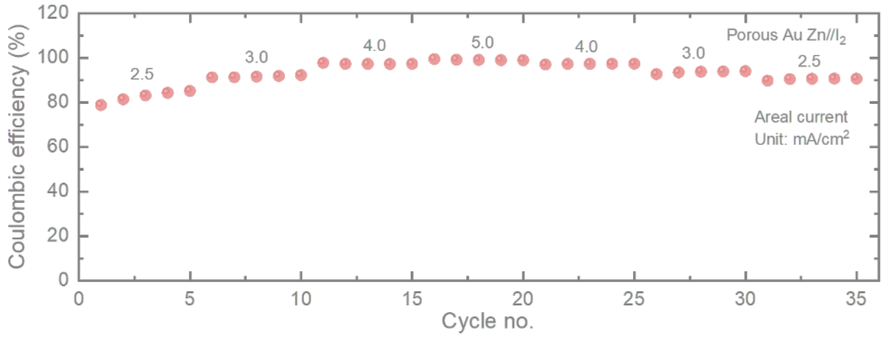

Figure S10. *Coulombic efficiency rate test of porous Au Zn//I_2 MB device across different areal currents, progressively increasing from 2.5 mA/cm² to 5 mA/cm², followed by a return to 2.5 mA/cm².*

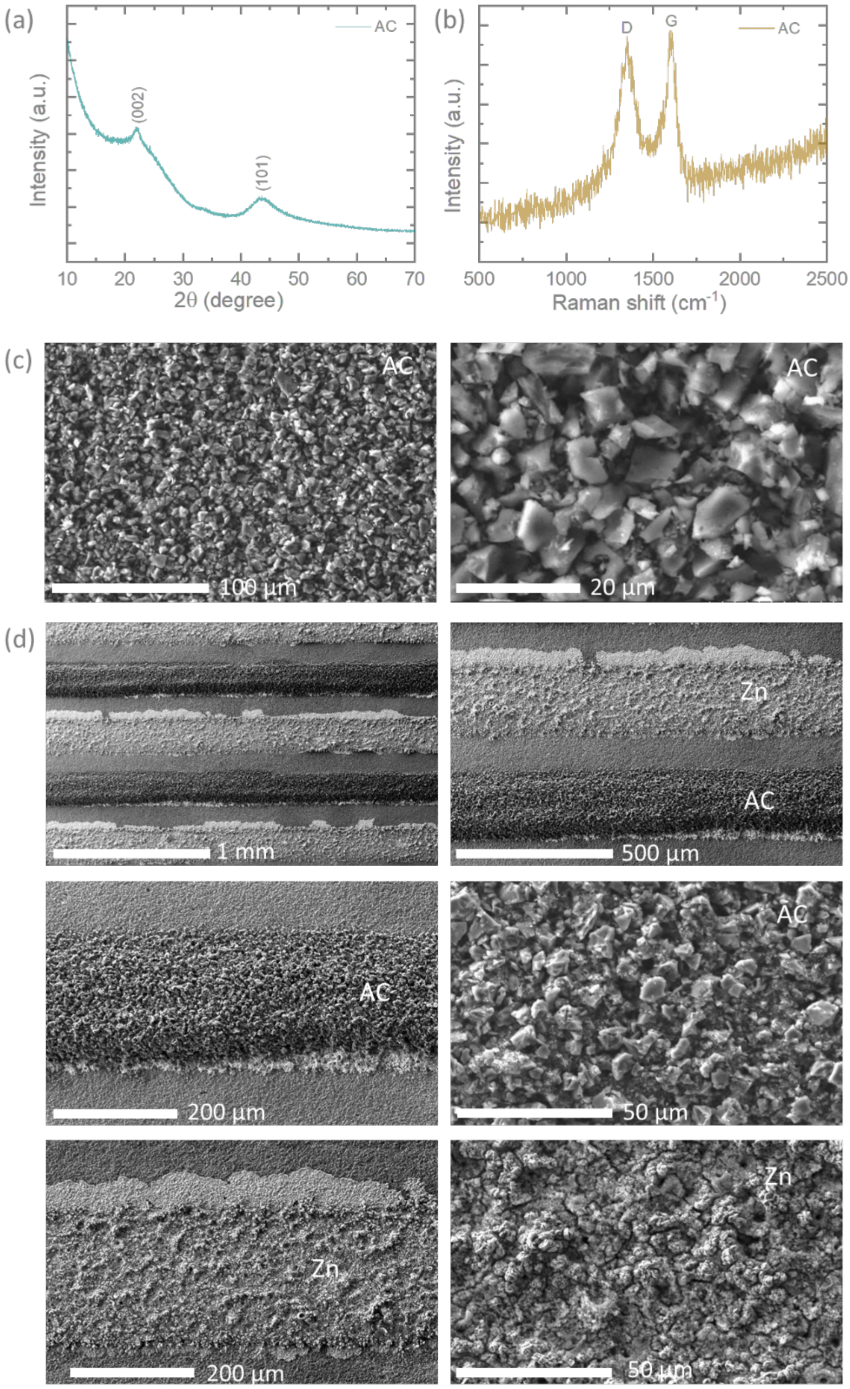

Figure S11. (a) XRD pattern (b) Raman spectrum and (c) SEM images of AC. (d) SEM images of AC and Zn loaded on porous Au IDEs.

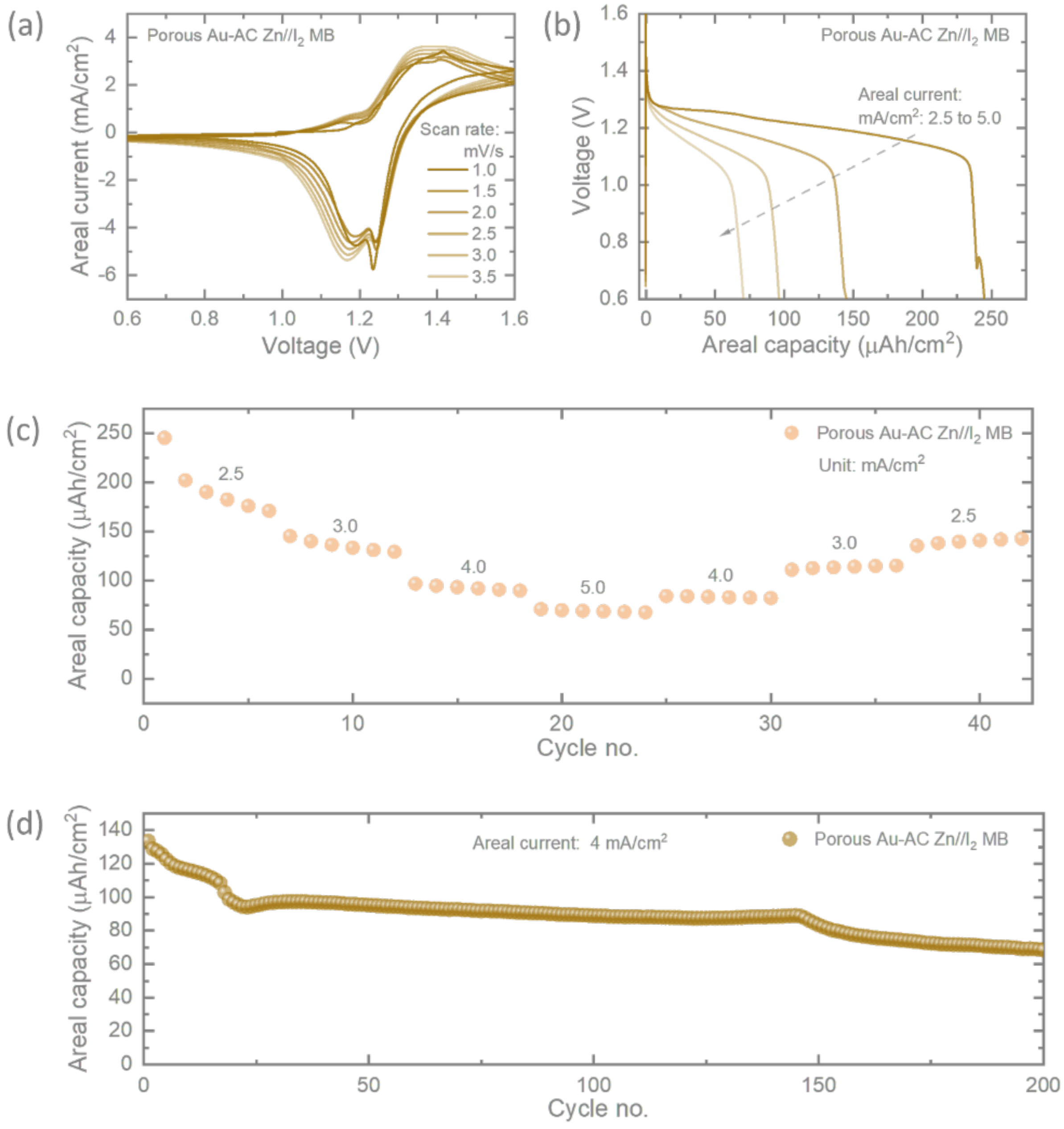

Figure S12. *(a) CV curves of porous Au-AC Zn//I*$_2$ *MB at different scan rates ranging from 1 to 3.5 mV/s. (b) GCD curves of porous Au-AC Zn//I*$_2$ *MB device at different areal currents ranging from 2.5 mA/cm*^{2} *to 5 mA/cm*^{2}*. (c) Rate test of porous Au-AC Zn//I*$_2$ *MB across different areal currents, progressively increasing from 2.5 mA/cm*^{2} *to 5 mA/cm*^{2}*, followed by a return to 2.5 mA/cm*^{2}*. (d) Long-term cycling performance of porous Au-AC Zn//I*$_2$ *MB device over 200 cycles at a fixed areal current of 4 mA cm*$^{-2}$*.*

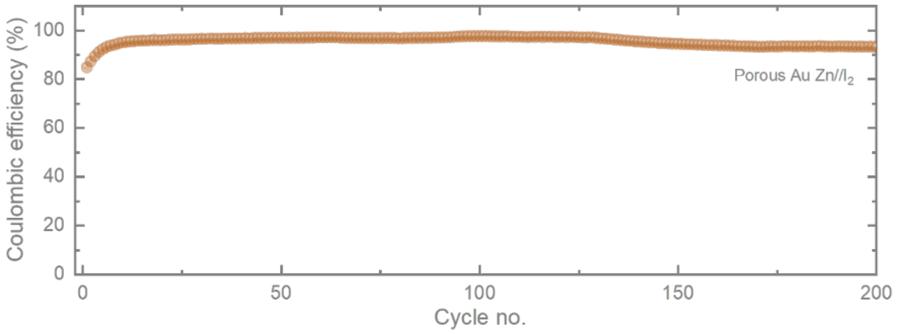

Figure S13. *Coulombic efficiency test of long-term cycles for porous Au Zn//I*$_2$ *MB device across at an areal current of 4 mA/cm*^{2}.

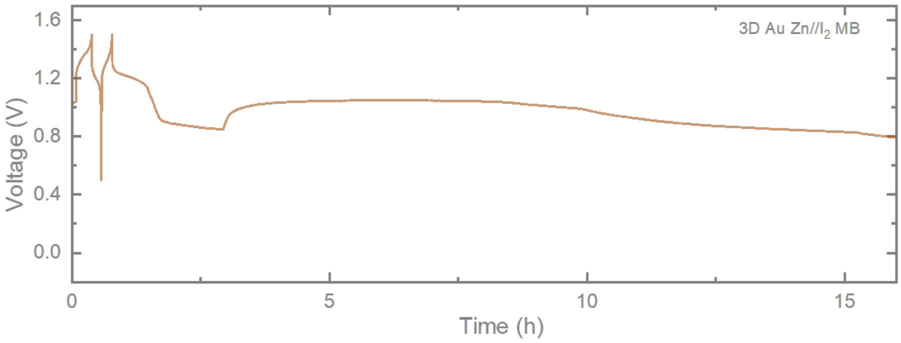

Figure S14. *Self-Discharge test of porous Au Zn//I*$_2$ *MB device*

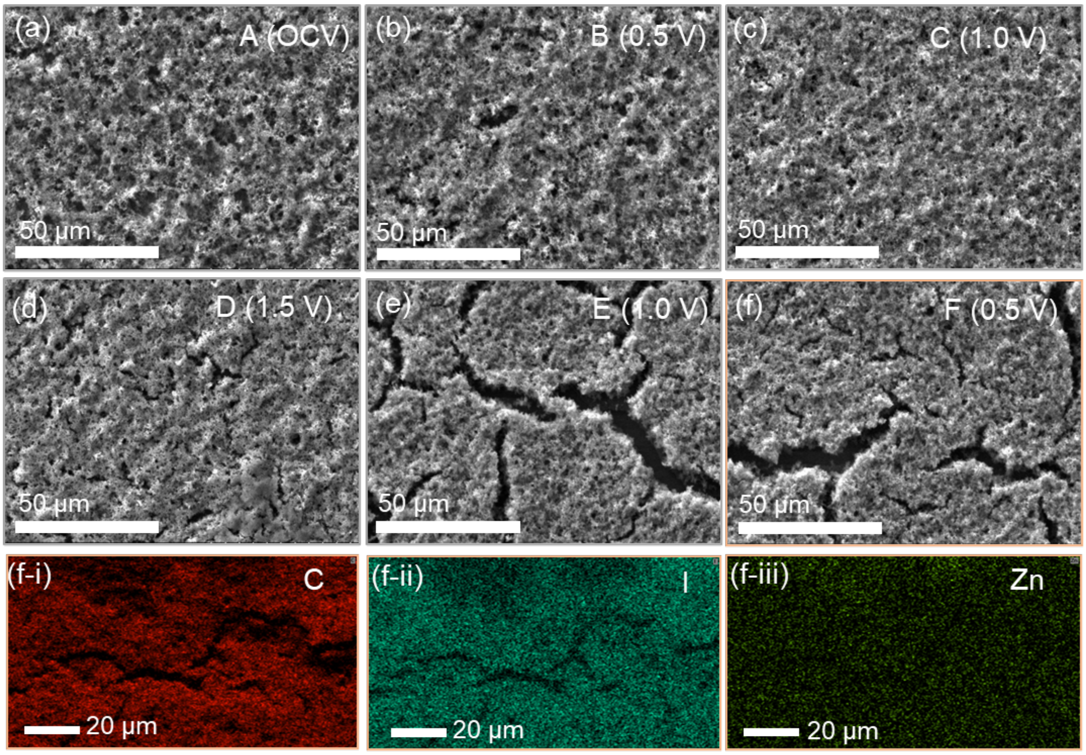

Figure S15. *(a-c) SEM images of PANI cathodes at OCV, A (0.5 V), and B (1.0 V) show nanowire-like morphology. (d-f) SEM images of PANI cathodes at SoC points C (1.5 V), D (1.0 V), and E (0.5 V) reveal the development of surface cracks, with minor cracks observed at C and more pronounced crack propagation at D and E. (f, i–iv) EDS elemental mapping of the PANI cathode at E (0.5 V) shows the distribution of C, Zn, and I elements*

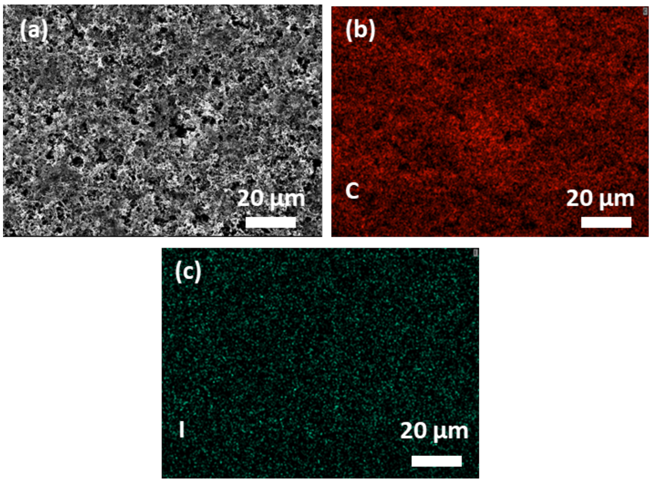

Figure S16. *(a) SEM images of PANI cathode at low magnification for state of charge OCV shows porous network structure (c, d) EDS elemental mapping of the PANI cathode at OCV state shows the distribution of C, and I elements.*

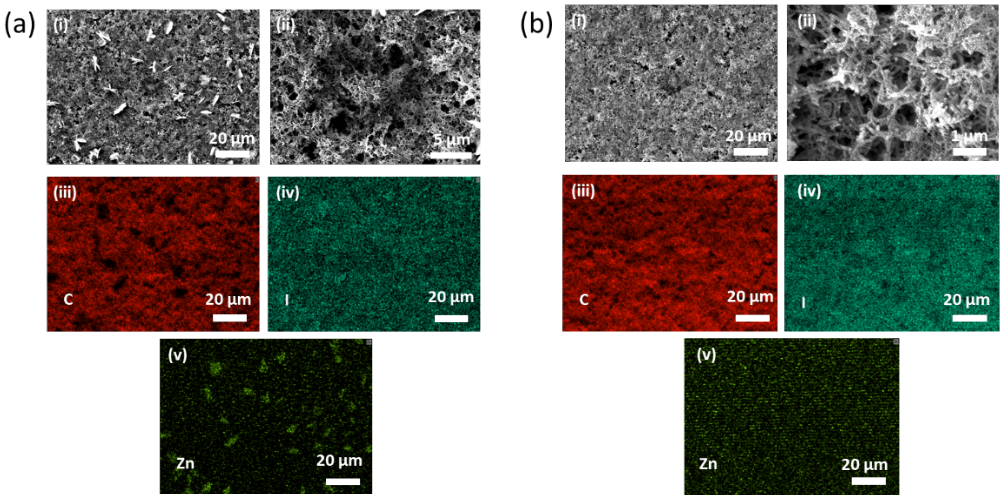

Figure S17. *a(i, ii) SEM images of PANI cathode at different magnification for state of charge A (0.5 V) shows porous network nanowire structure, a(iii, iv, v) EDS elemental mapping of the PANI cathode at A (0.5 V) state shows the uniform distribution of C, I and Zn elements. b(i,ii) SEM images of PANI cathode at different magnification for state of charge B (1.0 V) shows porous network nanowire structure, b(iii,iv,v) EDS elemental mapping of the PANI cathode at B (1.0 V) state shows the uniform distribution of C, I and Zn elements.*

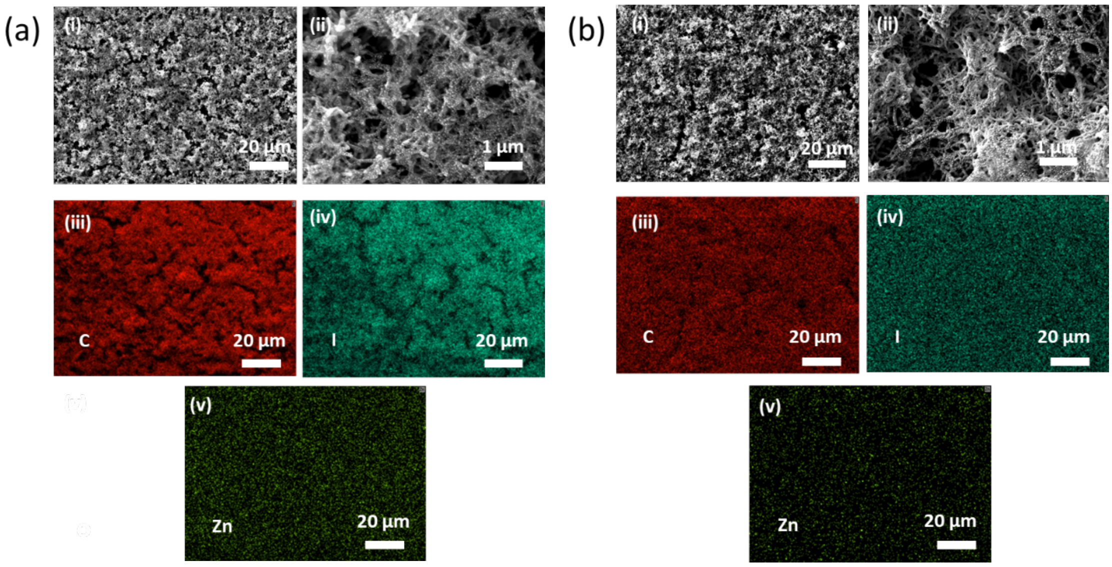

Figure S18. *a(i, ii) SEM images of PANI cathode at different magnification for state of charge C (1.5 V) shows porous network nanowire structure, a(iii, iv, v) EDS elemental mapping of the PANI cathode at C (1.5 V) state shows the uniform distribution of C, I and Zn elements. b(i,ii) SEM images of PANI cathode at different magnification for state of charge D (1.0 V) shows*

porous network nanowire structure, b(iii,iv,v) EDS elemental mapping of the PANI cathode at D (1.0 V) state shows the uniform distribution of C, I and Zn elements.

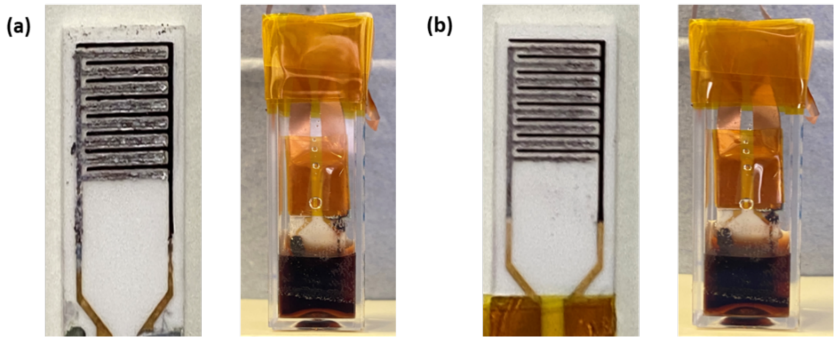

Figure S19. *Digital images of (a) flat Au Zn//I_2 MB and (b) porous Au Zn//I_2 MB devices after cycles.*

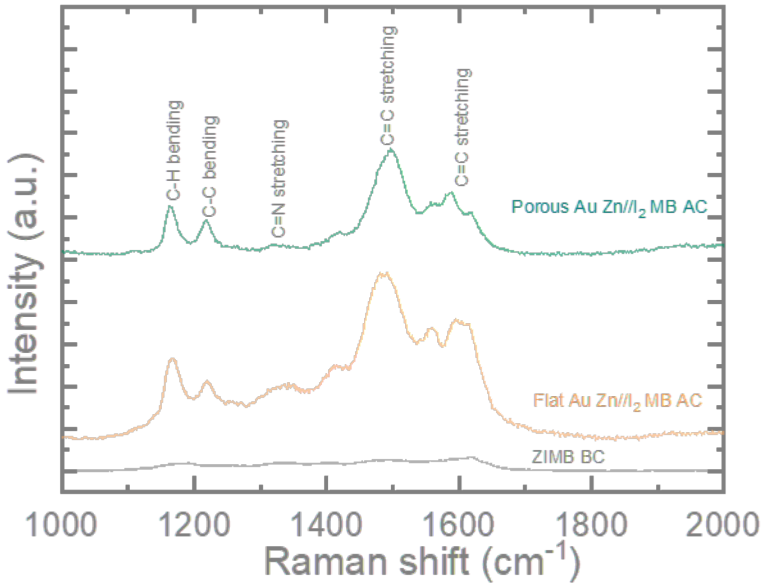

Figure S20. *Raman spectra comparing the pristine and cycled PANI cathodes after 200 cycles.*

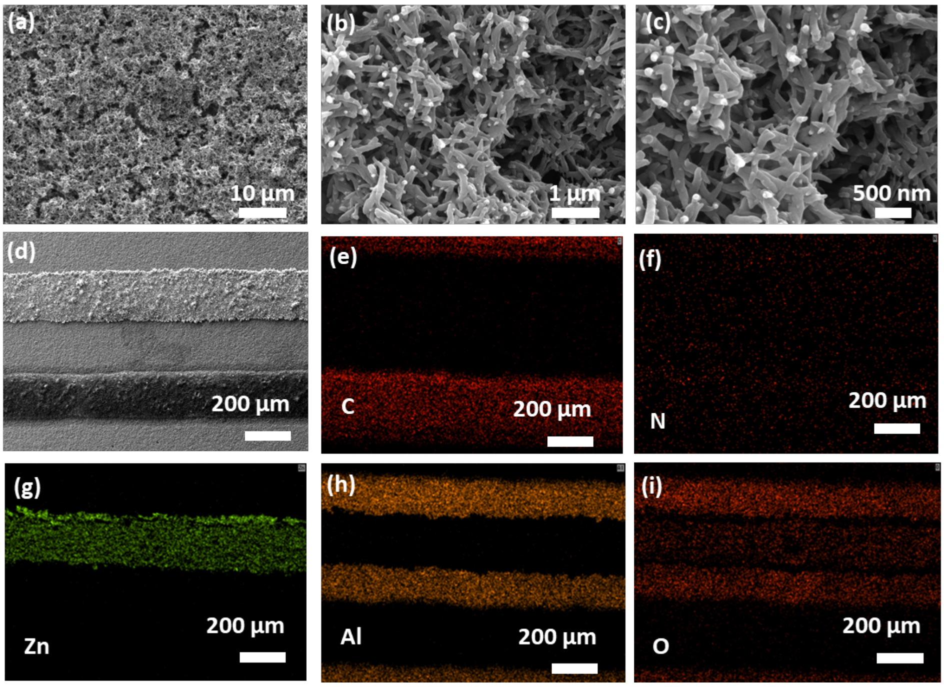

Figure S21. *(a-c) SEM images of ZIMB PANI cathode at different magnification after cycles shows presence of cracks on the surface and further magnified SEM images reveal that the PANI has a nanowire-like morphology with distinct porosities, (d) SEM images of ZIMB at low magnification show intact adhesion of the PANI cathode and Zn anode to the flat Au electrode. (e, f) EDS elemental mapping of the PANI cathode shows the distribution of C, and N elements, (g) EDS elemental mapping of the Zn anode shows the uniform distribution of Zn elements. (h, i) EDS elemental mapping of the ceramic substrate shows the distribution of Al, and O elements,*

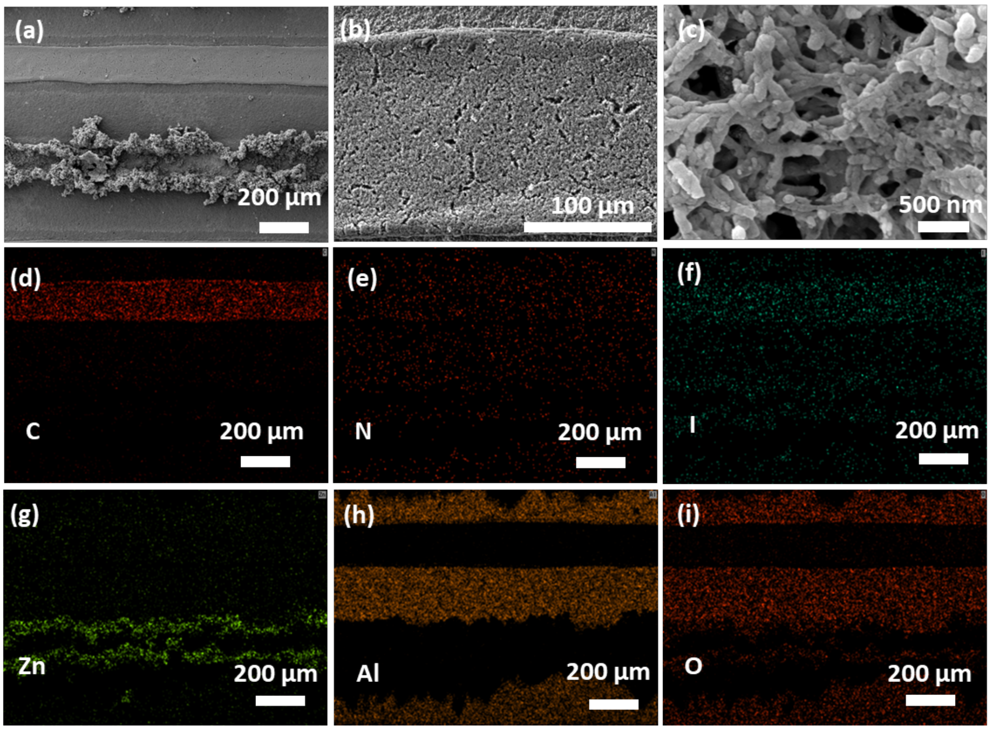

Figure S22. *(a) SEM images of flat Au Zn//I_2 MB after 200 cycles at low magnification show intact adhesion of the PANI cathode and Zn anode to the flat Au electrode. (b, c) SEM images of flat Au Zn//I_2 MB PANI cathode at different magnification after cycles shows presence of cracks on the surface and further magnified SEM images reveal that the PANI has a nanowire-like morphology with distinct porosities, (d, e) EDS elemental mapping of the PANI cathode shows the distribution of C, and N elements, (f) EDS elemental mapping of the device shows the distribution of I elements in both cathode and anode, (g) EDS elemental mapping of the Zn anode shows the uniform distribution of Zn elements. (h, i) EDS elemental mapping of the ceramic substrate shows the distribution of Al, and O elements.*

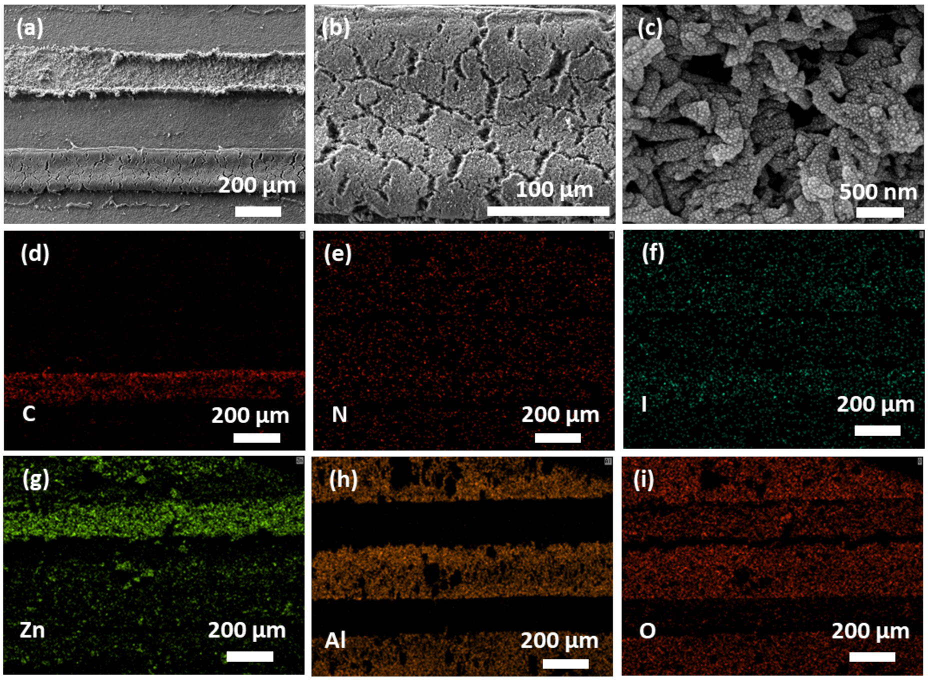

Figure S23. *(a) SEM images of porous Au Zn//I_2 MB after 200 cycles at low magnification show intact adhesion of the PANI cathode and Zn anode to the flat Au electrode. (b, c) SEM images of porous Au Zn//I_2 MB PANI cathode at different magnification after 200 cycles shows presence of cracks on the surface and further magnified SEM images reveal that the PANI has a nanowire-like morphology with distinct porosities, (d, e) EDS elemental mapping of the PANI cathode shows the distribution of C, and N elements, (f) EDS elemental mapping of the device shows the distribution of I elements in both cathode and anode, (g) EDS elemental mapping of the Zn anode shows the uniform distribution of Zn elements. (h, i) EDS elemental mapping of the ceramic substrate shows the distribution of Al, and O elements.*

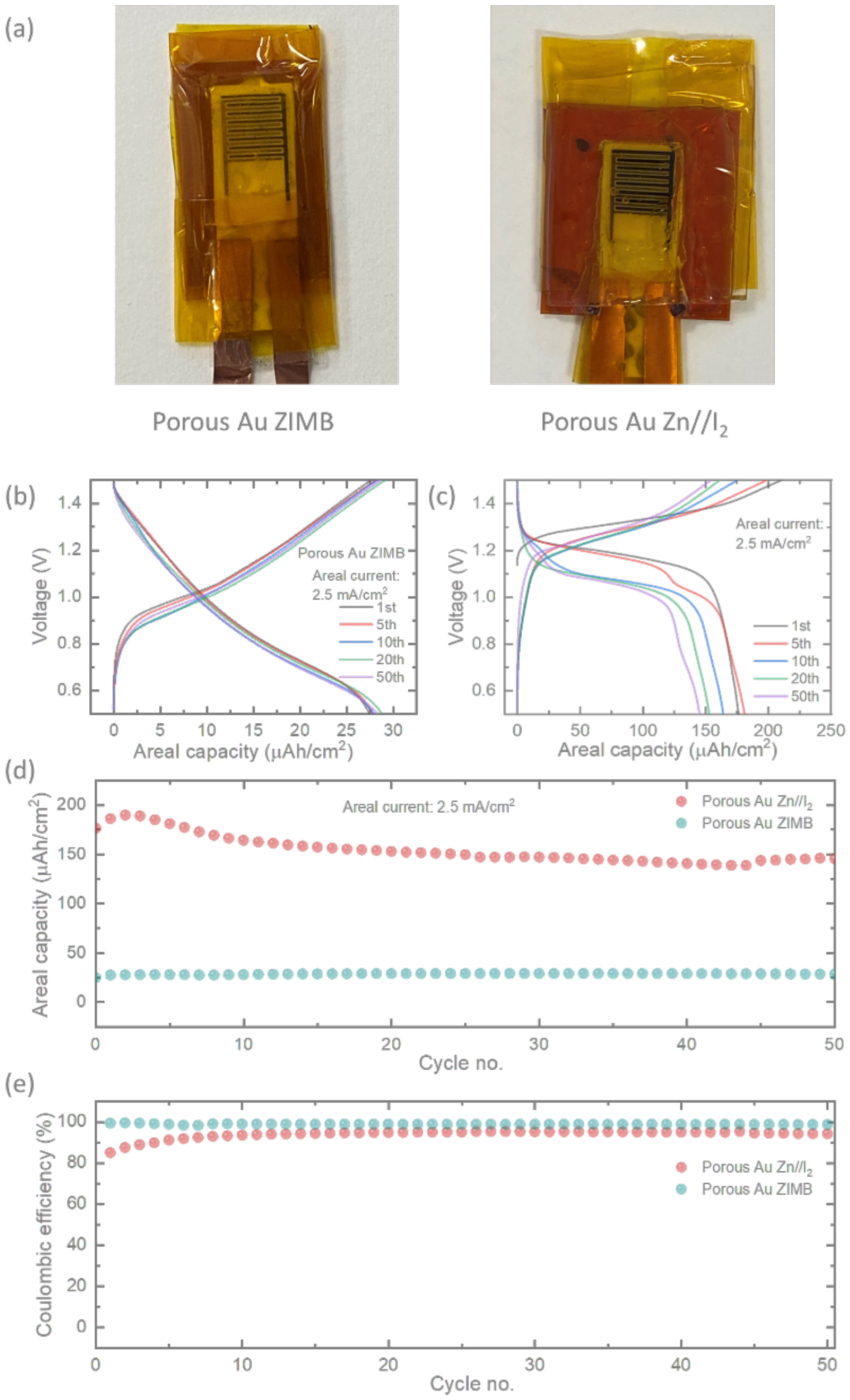

Figure S24. *Digital images of packaged devices: (a) porous Au ZIMB and (b) porous Au Zn//I*$_2$ *MB with 120 μL of gel electrolyte. GCD curves at an areal current density of 2.5 mA cm*$^{-2}$ *for (c) porous Au ZIMB and (d) porous Au Zn//I*$_2$*. (e) Comparison of areal capacity and (f) coulombic efficiency over 50 cycles at 2.5 mA cm*$^{-2}$*.*

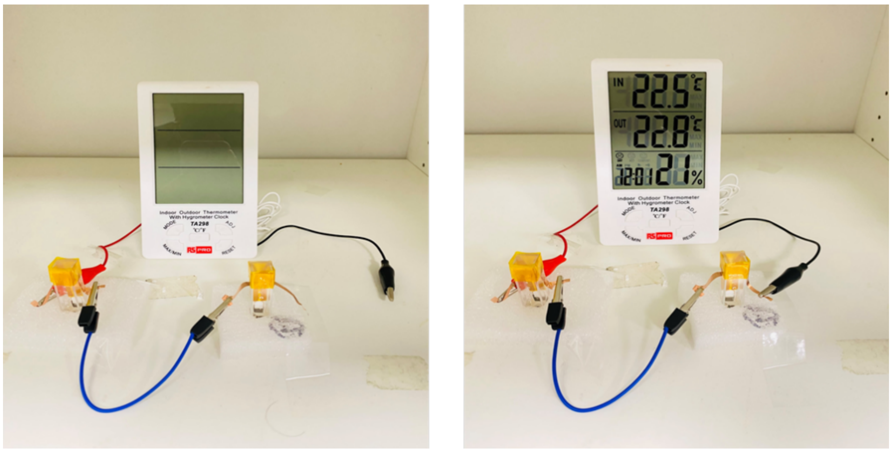

Figure S25. *Powering an indoor-outdoor thermometer with a hygrometer clock using our dual porous Au Zn//I_2 MB connected series.*

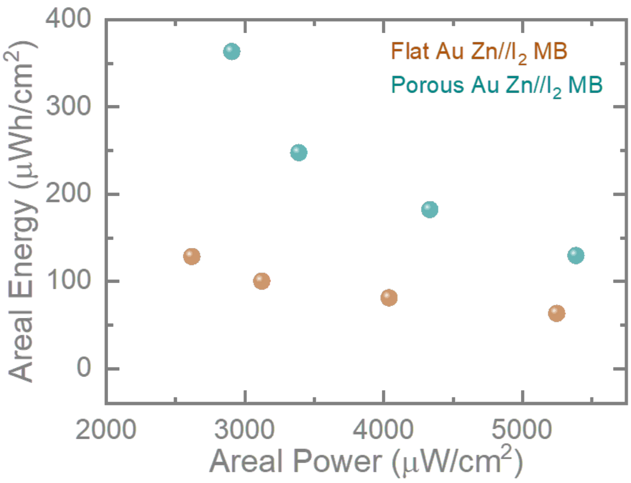

Figure S26. *Areal energy vs Areal power plot of flat Au Zn//I_2 MB and porous Au Zn//I_2 MB at areal current of 2.5 mA/cm^2 to 5 mA/cm^2.*

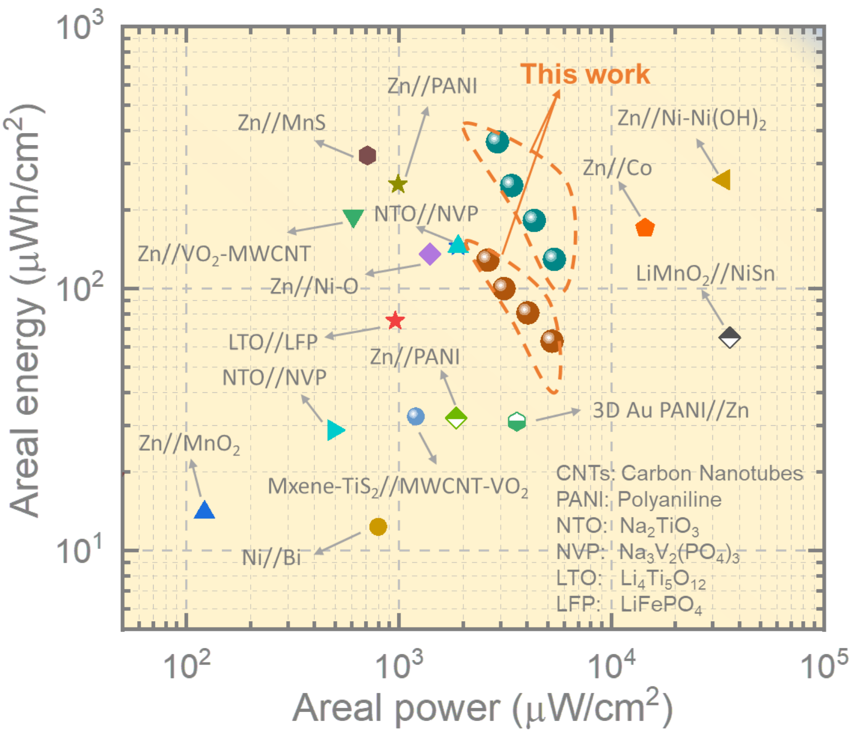

Figure S27. Ragone plot comparing the areal energy and areal power performance of the flat Au Zn//I_2 MB and porous Au Zn//I_2 MB with previously reported various types of MBs: Zn//MnO_2,[9] Zn//VO_2-MWCNT,[10] Zn//Ni-Co,[11] Zn//Ni-Ni$(OH)_2$,[12] NVP//NVP,[13] Zn//MnS,[14] Zn//PANI,[15] Co//Zn,[16] MXene-TiS_2/MWCNT-VO_2,[17] Zn/PANI,[18] $LiMnO_2$//Ni-Sn,[19] LFP//LTO,[20] NTO//NVP,[21] 3D Au PANI//Zn,[22] $LiMn_2O_4$//$Li_4Mn_5O_{12}$,[23] Ni//Bi,[24] PANI//Zn.[25]

References:

[1] L.J. Bartolotti, K. Flurchick, "An introduction to density functional theory," Rev Comput Chem (1996): 187–216.

[2] N. Argaman, G. Makov, "Density functional theory: An introduction," Am J Phys 68 (2000): 69–79.

[3] N.M. Harrison, "An introduction to density functional theory," Nato Sci Ser Sub Ser III Comput Syst Sci 187 (2003): 45–70.

[4] G. Kresse, J. Furthmüller, "Efficiency of ab-initio total energy calculations for metals and semiconductors using a plane-wave basis set," Comput Mater Sci 6 (1996): 15–50.

[5] G. Kresse, J. Furthmüller, "Efficient iterative schemes for ab initio total-energy calculations using a plane-wave basis set," Phys Rev B 54 (1996): 11169.

[6] J.P. Perdew, K. Burke, M. Ernzerhof, "Generalized gradient approximation made simple," Phys Rev Lett 77 (1996): 3865.

[7] P.E. Blöchl, "Projector augmented-wave method," Phys Rev B 50 (1994): 17953.

[8] S. Grimme, J. Antony, S. Ehrlich, H. Krieg, "A consistent and accurate ab initio parametrization of density functional dispersion correction (DFT-D) for the 94 elements H-Pu," J Chem Phys 132 (2010).

[9] X. Wang, S. Zheng, F. Zhou, J. Qin, X. Shi, S. Wang, C. Sun, X. Bao, Z.S. Wu, "Scalable fabrication of printed Zn//MnO_2 planar micro-batteries with high volumetric energy density and exceptional safety," Natl Sci Rev 7 (2020): 64–72.

[10] J. Shi, S. Wang, X. Chen, Z. Chen, X. Du, T. Ni, Q. Wang, L. Ruan, W. Zeng, Z. Huang, "An Ultrahigh Energy Density Quasi-Solid-State Zinc Ion Microbattery with Excellent Flexibility and Thermostability," Adv Energy Mater 9 (2019): 1–9.

[11] Z. Tian, Z. Sun, Y. Shao, L. Gao, R. Huang, Y. Shao, R.B. Kaner, J. Sun, "Ultrafast rechargeable Zn micro-batteries endowing a wearable solar charging system with high overall efficiency," Energy Environ Sci 14 (2021): 1602–1611.

[12] Z. Hao, L. Xu, Q. Liu, W. Yang, X. Liao, J. Meng, X. Hong, L. He, L. Mai, "On-Chip Ni–Zn Microbattery Based on Hierarchical Ordered Porous Ni@$Ni(OH)_2$ Microelectrode with Ultrafast Ion and Electron Transport Kinetics," Adv Funct Mater 29 (2019): 1–9.

[13] X. Wang, H. Huang, F. Zhou, P. Das, P. Wen, S. Zheng, P. Lu, Y. Yu, Z.S. Wu, "High-voltage aqueous planar symmetric sodium ion micro-batteries with superior performance at low-temperature of −40 °C," Nano Energy 82 (2021): 105688.

[14] K. Jiang, Z. Zhou, X. Wen, Q. Weng, "Fabrications of High-Performance Planar Zinc-Ion Microbatteries by Engraved Soft Templates," Small 17 (2021): 1–8.

[15] R. Li, L. Li, R. Jia, K. Jiang, G. Shen, D. Chen, "A Flexible Concentric Circle Structured Zinc-Ion Micro-Battery with Electrodeposited Electrodes," Small Methods 4 (2020): 1–9.

[16] Y. Wang, X. Hong, Y. Guo, Y. Zhao, X. Liao, X. Liu, Q. Li, L. He, L. Mai, "Wearable Textile-Based Co−Zn Alkaline Microbattery with High Energy Density and Excellent Reliability," Small 16 (2020): 1–9.

[17] B. Zhao, S. Wang, Q. Yu, Q. Wang, M. Wang, T. Ni, L. Ruan, W. Zeng, "A flexible, heat-resistant and self-healable “rocking-chair” zinc ion microbattery based on MXene-TiS2 (de)intercalation anode," J Power Sources 504 (2021): 230076.

[18] S. Bi, F. Wan, S. Huang, X. Wang, Z. Niu, "A Flexible Quasi-Solid-State Bifunctional Device with Zinc-Ion Microbattery and Photodetector," ChemElectroChem 6 (2019): 3933–3939.

[19] H. Ning, J.H. Pikul, R. Zhang, X. Li, S. Xu, J. Wang, J.A. Rogers, W.P. King, P. V. Braun, "Holographic patterning of high-performance on-chip 3D lithium-ion microbatteries," Proc Natl Acad Sci U S A 112 (2015): 6573–6578.

[20] S. Zheng, Z.S. Wu, F. Zhou, X. Wang, J. Ma, C. Liu, Y.B. He, X. Bao, "All-solid-state planar integrated lithium ion micro-batteries with extraordinary flexibility and high-temperature performance," Nano Energy 51 (2018): 613–620.

[21] S. Zheng, H. Huang, Y. Dong, S. Wang, F. Zhou, J. Qin, C. Sun, Y. Yu, Z.S. Wu, X. Bao, "Ionogel-based sodium ion micro-batteries with a 3D Na-ion diffusion

mechanism enable ultrahigh rate capability," Energy Environ Sci 13 (2020): 821–829.

[22] N. Naresh, Y. Zhu, J. Luo, Y. Fan, T. Wang, K. Raju, M. De, I.P. Parkin, B.D. Boruah, "Advanced 3D Micro-Electrodes for On-Chip Zinc-Ion," Adv. Funct. Mater 2413777 (2025): 1–10.

[23] M. Kotobuki, Y. Suzuki, H. Munakata, K. Kanamura, Y. Sato, K. Yamamoto, T. Yoshida, "Effect of sol composition on solid electrode/solid electrolyte interface for all-solid-state lithium ion battery," Electrochim Acta 56 (2011): 1023–1029.

[24] L. He, T. Hong, X. Hong, X. Liao, Y. Chen, W. Zhang, H. Liu, W. Luo, L. Mai, "Ultrastable High-Energy On-Chip Nickel–Bismuth Microbattery Powered by Crystalline Bi Anode and Ni–Co Hydroxide Cathode," Energy Technol 7 (2019): 1–7.

[25] N. Naresh, Y. Fan, Y. Zhu, T. Wang, S. Li, I.P. Parkin, "3D Porous Metal-Scaffold Interdigitated Micro-Electrodes for High-Performance On-Chip Energy Storage Systems," Adv. Funct. Mater 2507537 (2025): 1–11.
